# Relaxation of magnetization in Dy_2_ and Tb_2_ dimers with single-electron lanthanide–lanthanide bonds: the effect of cage perturbation and its ultimate symmetry

**DOI:** 10.1039/d6sc04567g

**Published:** 2026-08-19

**Authors:** Matheus Felipe de Souza Barbosa, Wei Yang, Yaofeng Wang, Georgios Velkos, Stanislav M. Avdoshenko, Fupin Liu, Alexey A. Popov

**Affiliations:** a Leibniz Institute for Solid State and Materials Research (IFW Dresden) Helmholtzstr. 20 01069 Dresden Germany s.avdoshenko@ifw-dresden.de a.popov@ifw-dresden.de; b Jiangsu Key Laboratory of New Power Batteries, Jiangsu Collaborative Innovation Center of Biomedical Functional Materials, School of Chemistry and Materials Science, Nanjing Normal University Nanjing 210023 China liu_fupin@nnu.edu.cn

## Abstract

Covalent bonding between lanthanide ions dramatically enhances exchange interactions between their magnetic moments and leads to fascinating magnetic properties. Particularly attractive for this goal are single-electron lanthanide–lanthanide bonds, which were first realized in metal dimers encapsulated in endohedral metallofullerenes. So far, stabilization of the single-electron bond in fullerenes required modification of the carbon cage by replacing one carbon with nitrogen or by an exohedral attachment of a radical group, which inevitably perturbed the cage symmetry and thereby affected the magnetic anisotropy. Here, we report on the isolation of [M_2_@*I*_h_-C_80_]^−^ anions (M = Tb, Dy) with single-electron M–M bonds, for which we developed a chromatography-free separation protocol. Thorough investigation of their dynamic magnetic properties and relaxation mechanisms in comparison to those of M_2_@C_80_(CF_3_), M_2_@C_80_(CH_2_Ph), and M_2_@C_79_N showed that highly symmetric [M_2_@C_80_]^−^ anions exhibit the highest blocking temperatures in the series. Magnetization relaxation parameters depend considerably on the mode of the cage functionalization, and Dy_2_ is more susceptible to the variation of its environment than Tb_2_. Our work opens the way to capture elusive anions of endohedral metallofullerenes without perturbing their cage symmetry, facilitating deep investigation of these fascinating molecules.

## Abstract 2

Anions of Dy_2_@C_80_ and Tb_2_@C_80_ are isolated in the solid state and the influence of the fullerene host on the single-molecule magnetism properties of Dy and Tb dimetallofullerenes with single-electron metal–metal bonds is studied.

## Graphical abstract

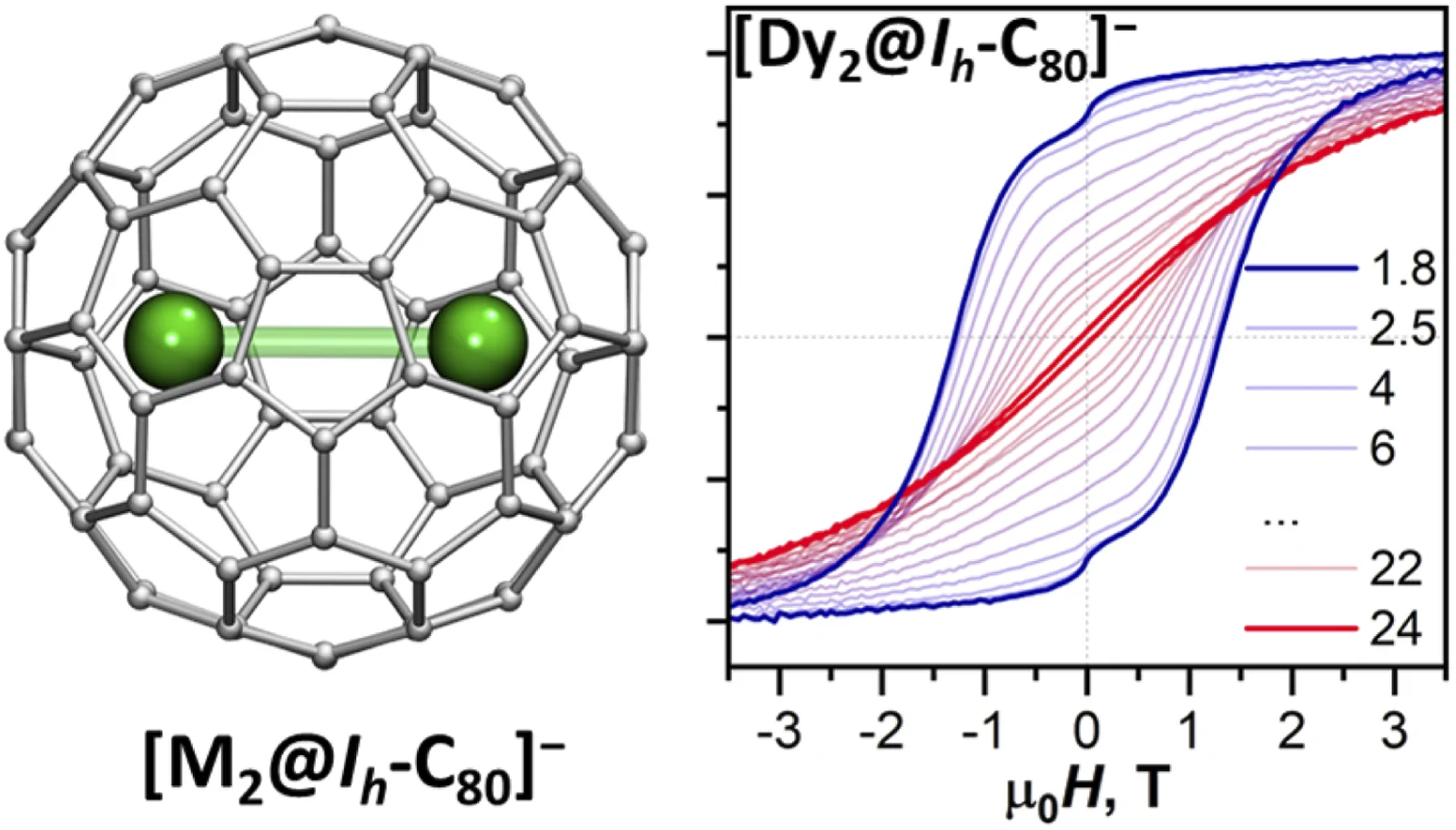

## Introduction

Single-electron metal–metal (1e–2M) bonds between rare-earth elements were first discovered in metal dimers encapsulated in fullerenes (known as dimetallofullerenes or di-EMFs)^1–18^ and later obtained in the (Cp^*i*^Pr_5_)_2_Ln_2_I_3_ complexes.^19^ In di-EMFs, this bonding motif emerges when the rare-earth dimer is encapsulated inside a carbon cage and transfers its valence electrons to the host. The degree of the electron transfer naturally depends on the energy matching between molecular orbital levels of the metal dimer and the fullerene.^14,20,21^ For lanthanide dimers starting at least from Nd,^11^ the energy of the *σ*^2^ metal–metal bonding MO appears too low in comparison to the empty cage MOs, leading to only one electron being transferred from this orbital to the fullerene. Di-EMFs of such lanthanides still have one unpaired electron left behind on the M–M bonding orbital inside the fullerene, resulting in a bonding situation described as the 1e–2M bond. Covalently bonded heavy-lanthanide dimers exhibit unprecedented magnetic properties,^14,22,23^ including the highest blocking temperature of magnetization among single-molecule magnets (SMMs) and a very broad magnetic hysteresis.^9,19^ A particular advantage of fullerenes as hosts is the protection of metal dimers from the environment, which provides the air and thermal stability required in practical applications and enables the growth of thin films by evaporation,^24^ electrospray deposition,^25,26^ or self-assembly from solution.^27^ Excellent SMM properties of covalently bonded lanthanide dimers stem from a combination of giant exchange coupling and single-ion magnetic anisotropy, the latter being determined by the coordination environment. In di-EMFs, the anisotropy is driven by the metal–metal bonding density and coordination to the fullerene cage.^10,28^ The role of the cage beyond being merely a container remained obscure, but attracted considerable interest recently,^17,29,30^ especially after the discovery of the superior SMM performance of Dy@C_81_N, which demonstrated that the slow relaxation of magnetization in a single lanthanide ion can be induced by a carbon cage alone.^31^

**Fig. 1:**
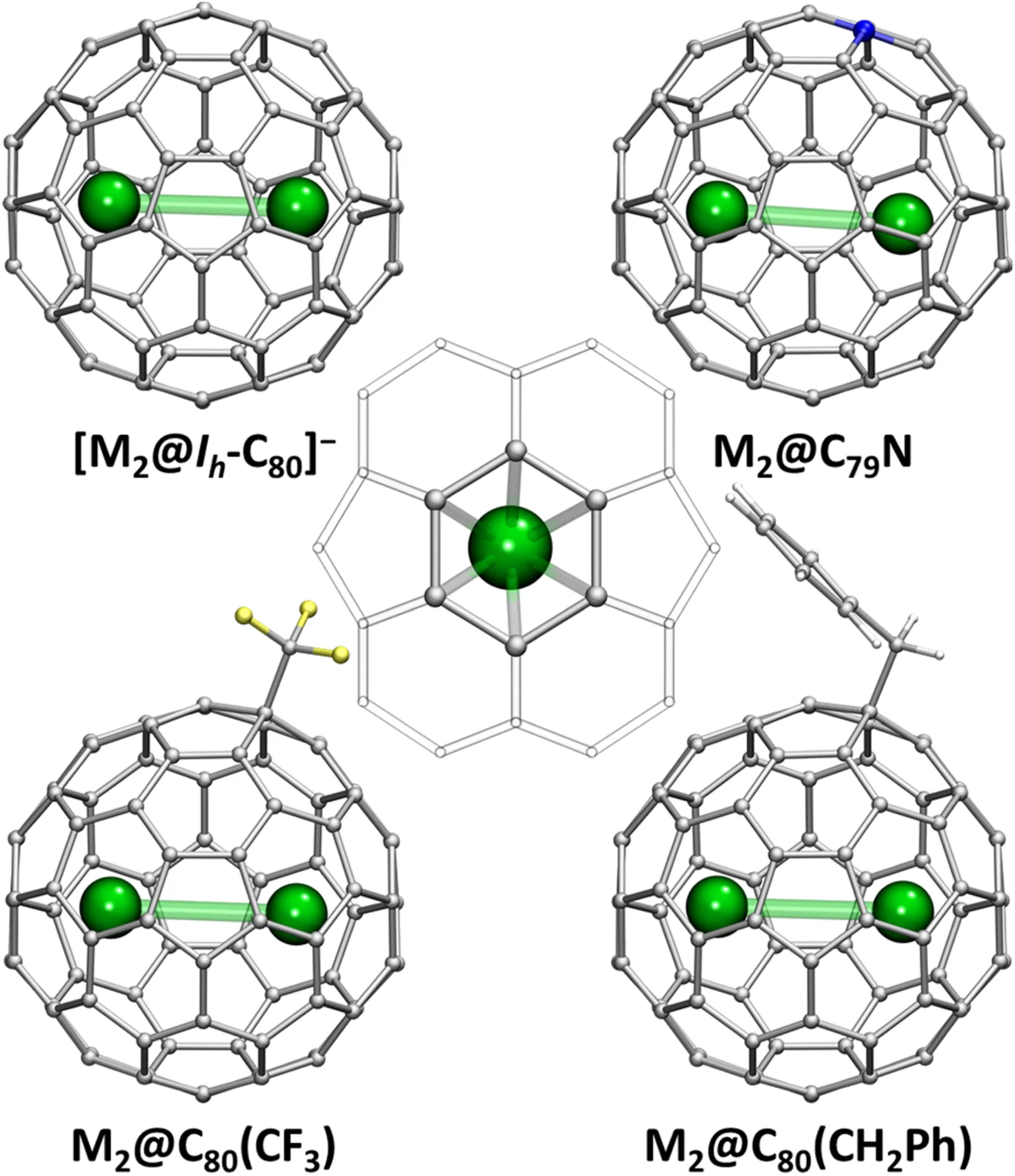
Stabilized forms of M_2_@C_80_ (M = Tb, Dy) with an *I*_h_-symmetric fullerene cage: anion [M_2_@C_80_]^−^, azafullerene M_2_@C_79_N, trifluoromethyl (CF_3_) and benzyl (CH_2_Ph) monoadducts; all molecules feature a single-electron M–M bond and a closed-shell electronic structure of the carbon cage. The inset in the center shows *η*^6^ metal-cage coordination in [M_2_@C_80_]^−^: the metal atom is slightly displaced towards one of the pentagon/hexagon edges of the coordinated hexagon; similar coordination is found in other molecules.

Most of the reported di-EMFs with 1e–2M bonds are based on the highly symmetric *I*_h_-C_80_ cage. However, unless the endohedral dimer is heterometallic,^15,32–34^ the fullerene cage in M_2_@C_80_ with a 1e–2M bond has an open-shell electronic structure^35^ and requires stabilization either by replacing one carbon with nitrogen^2,3^ or by an exohedral attachment of a radical group (CF_3_,^11,12^ CH_2_Ph,^6,8–10^ or triazinyl radical^7^). Thus, the *I*_h_-C_80_ cage in M_2_@C_79_N, M_2_@C_80_(CF_3_), and M_2_@C_80_(CH_2_Ph) has a defect in the form of a nitrogen or a C-sp^3^ atom (Fig. 1), which inevitably reduces the symmetry to *C*_s_ and thereby affects the magnetic anisotropy. The sensitivity of endohedral metals to these “defects” is clear from variable-temperature single-crystal X-ray diffraction (SC-XRD)^9^ and computational studies,^1,10,12^ which demonstrated that metal dimers tend to avoid N or C-sp^3^ sites.

Another well-known approach to stabilize the open-shell EMF is to reduce it to the anionic state. Widely used redox-extraction of EMFs by hot dimethylformamide (DMF) is based on this principle, since partial thermal decomposition of DMF during extraction produces amines, which reduce open-shell EMFs to more kinetically stable EMF anions.^36^ The DMF-extracted [M_2_@*I*_h_-C_80_]^−^ anion in a mixture with other EMF anions served as a precursor in the synthesis of M_2_@C_80_(CF_3_) and M_2_@C_80_(CH_2_Ph), but has not been isolated and studied on its own for anisotropic lanthanides. Only isolation of [Gd_2_@C_80_]^−^ and its EPR study were reported in one conference abstract.^37^ The EMF anions are scarcely studied, and only a couple of them were described in the solid state.^38–42^ In this work, we developed a redox-based procedure for a facile isolation of [Dy_2_@*I*_h_-C_80_]^−^ and [Tb_2_@*I*_h_-C_80_]^−^ anions. This allowed us to study the magnetic relaxation of Dy_2_ and Tb_2_ dimers in a symmetric environment and disclose the role of the fullerene cage by comparing the SMM behavior of [M_2_@C_80_]^−^ to that of M_2_@C_79_N, M_2_@C_80_(CF_3_) and M_2_@C_80_(CH_2_Ph) (Fig. 1).

## Results and discussion

### Synthesis and isolation of EMFs

EMF-containing soot was obtained by the arc-discharge evaporation of graphite rods filled with a metal oxide (Dy_2_O_3_ or Tb_4_O_7_) in a 180 mbar He atmosphere with addition of 10 mbar N_2_. The soot was first extracted by CS_2_, which dissolved major empty fullerenes (C_60_, C_70_, *etc.*) and classical metallofullerenes (mainly M@C_2*n*_, M_3_N@C_2*n*_, and M_2_C_2_@C_2*n*_). The soot left after CS_2_ extraction was then extracted with boiling dimethylformamide (DMF), which produced a DMF solution of fullerene anions.

#### Isolation of Dy_2_@C_79_N

With the very low percentage of Dy_2_@C_79_N in the CS_2_ extract, its conventional isolation by high-performance liquid chromatography (HPLC) was found to be impractical at this stage. Instead, we first applied the SAFA (stir-and-filter approach), developed by Stevenson *et al.*^43,44^ The fullerene extract was dissolved in toluene and reacted with dry diamino-functionalized silica gel (DASG). In this process, empty fullerenes and some EMFs, including Dy_2_@C_79_N, were trapped by DASG, while slowly-reacting Dy_3_N@C_2*n*_ mainly remained in solution (Fig. S1). The key to the isolation of Dy_2_@C_79_N is the reversible uptake of EMFs by DASG.^43^ Washing the spent DASG with CS_2_ released EMFs, including Dy_2_@C_79_N, while empty fullerenes bind to DASG more strongly and remain trapped. Pure Dy_2_@C_79_N was then isolated from other EMFs using straightforward HPLC steps. Details of HPLC separation and mass spectrometry (MS) characterization are shown in the SI (Fig. S2).

#### Synthesis of Dy_2_@*I*_h_-C_80_(CF_3_)

Dy_2_@*I*_h_-C_80_(CF_3_) was synthesized using the electrophilic trifluoromethylation procedure developed by us earlier for the synthesis of Tb_2_@*I*_h_-C_80_(CF_3_).^12^ In brief, a mixture of Dy-EMF anions, extracted by DMF, was reacted with Umemoto Reagent II^45^ at room temperature. DMF was then removed under reduced pressure, and the reaction products were dissolved in toluene and separated by HPLC. At the optimized reagent ratio, Umemoto Reagent II shows high selectivity towards [Dy_2_@*I*_h_-C_80_]^−^, yielding Dy_2_@*I*_h_-C_80_(CF_3_) as the main product. Details of the reaction, HPLC separation and MS characterization of Dy_2_@*I*_h_-C_80_(CF_3_) are shown in the SI (Fig. S3 and S4).

#### Isolation of [M_2_@*I*_h_-C_80_]^−^

**Fig. 2:**
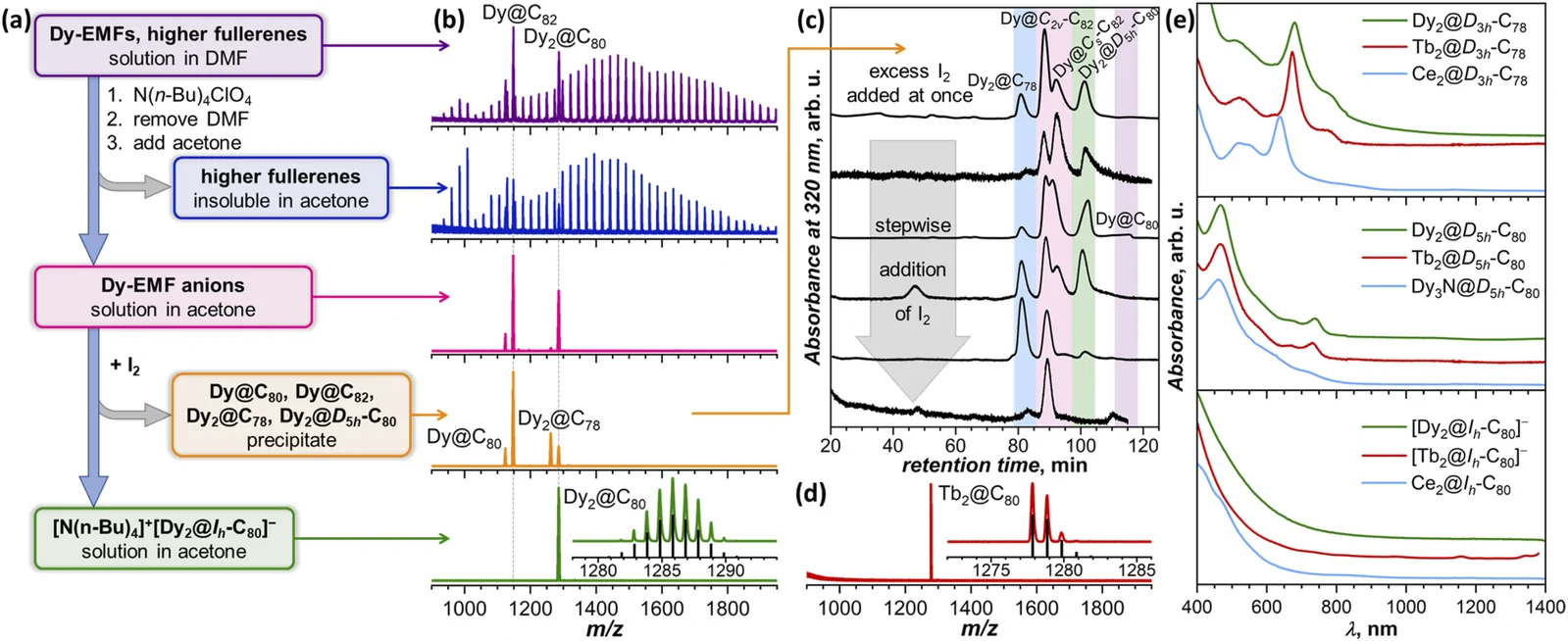
(a) Separation scheme of the Dy-EMF extract in DMF using oxidation with iodine. (b) Mass spectra of the intermediate mixture and isolated [Dy_2_@*I*_h_-C_80_]^−^ (the inset shows experimental and calculated isotopic patterns). (c) HPLC traces of Dy-EMFs precipitated by reaction with I_2_, the upper trace is for the reaction with all I_2_ added at once, and the lower traces are for the stepwise addition of I_2_ (eluent: toluene, Buckyprep column). (d) Mass spectrum of isolated [Tb_2_@*I*_h_-C_80_]^−^. (e) Vis-NIR absorption spectra of isolated Dy_2_@C_2*n*_ and Tb_2_@C_2*n*_ (neutrals – in toluene or CS_2_, anions – in acetone) and spectra of structurally characterized EMFs with known fullerene cages are shown in light blue.

To obtain [Dy_2_@*I*_h_-C_80_]^−^, we developed a strategy utilizing different redox properties of Dy-EMFs (Fig. 2a). Following Ref. 46, we added N(*n*-Bu)_4_ClO_4_ salt to the DMF solution to stabilize EMF anions with [N(*n*-Bu)_4_]^+^ counterions and then evaporated DMF under reduced pressure. Anionic salts of EMFs were then dissolved in acetone, in which neutral empty fullerenes are not soluble (see Fig. 2b for mass spectra). MS analysis of the acetone solution showed the presence of Dy@C_80_, Dy@C_82_, Dy_2_@C_78_, and Dy_2_@C_80_. The molecular structure of Dy@C_80_ with the *C*_2v_(5) cage isomer was recently determined by Yang *et al.*^47^ Dy@C_82_^−^ in the DMF extract is represented by three main cage isomers: *C*_s_(6), *C*_2v_(9), and the recently found *C*_3v_(8).^29^ The *D*_3h_(5) cage isomer is common for M_2_@C_78_,^48,49^ and we assigned this cage to Dy_2_@C_78_ (*vide infra*). Two isomers, minor *D*_5h_(6) and major *I*_h_(7), are typical for M_2_@C_80_ with trivalent lanthanides.^11,50^

While EMF anions show considerable solubility in acetone, neutral fullerenes are not soluble in this solvent. Therefore, if anions are oxidized to the neutral form, they immediately precipitate and, in principle, can be dissolved in a non-polar solvent and separated by HPLC. However, our attempts to oxidize the whole mixture of Dy-EMF anions by dichloroacetic acid^46^ gave a precipitate, which was not soluble in standard fullerene solvents CS_2_ and toluene. We therefore considered if a mild oxidant can be used for a partial separation of Dy-EMFs. To evaluate the feasibility of this approach, DFT calculations were performed for neutral and anionic states of Dy-EMFs in the gas phase and in acetone solution using the Orca suite.^51^ The DFT survey showed that Dy_2_@*I*_h_-C_80_ has the highest electron affinity and the most positive reduction potential (*E*^0/−^), Dy@*C*_3v_(8)-C_82_ is the next in line with *E*^0/−^ at −0.03 V relative to Dy_2_@*I*_h_-C_80_, while *E*^0/−^ values of other Dy-EMFs span the range of −0.29 V to −0.47 V (Table 1 and S1). This order of potentials indicates that it may be possible to oxidize and precipitate all Dy-EMFs except for [Dy_2_@*I*_h_-C_80_]^−^ by using a suitable oxidant with matching redox potential, and the separation of [Dy_2_@*I*_h_-C_80_]^−^ from [Dy@*C*_3v_(8)-C_82_]^−^ with very similar oxidation potentials can be the main problem on this route.

**Table 1:** DFT-computed electron affinity of Dy-EMFs in the gas phase (eV) and relative reduction potentials in acetone (in V) a

EMF	EA (gas)	ΔEA(gas)	Δ*E*^0/−^(acetone)
Dy@*C*_s_(6)-C_82_	3.33	−0.47	−0.47
Dy_2_@*D*_5h_(6)-C_80_	3.42	−0.38	−0.37
Dy@*C*_2v_(9)-C_82_	3.44	−0.36	−0.36
Dy@*C*_2v_(5)-C_80_	3.49	−0.31	−0.30
Dy_2_@*D*_3h_(5)-C_78_	3.49	−0.31	−0.29
Dy@*C*_3v_(8)-C_82_	3.77	−0.03	−0.03
Dy_2_@*I*_h_(7)-C_80_	3.80	0.00	0.00

a Computations performed at the PBE/def2-TZVPP level with a 4f-in-core effective potential for Dy and C-PCM solvation correction for acetone; the values are in eV for EA and in V for reduction potentials Δ*E*^0/−^.

After testing reactions of EMF anions with several mild oxidants, we found that molecular iodine is most suitable for this goal. Earlier, the oxidation of Y and La-EMF anions by iodine with formation of neutral EMFs was also noted by Kareev *et al.* based on the electrospray MS analysis.^52^ Addition of excess I_2_ to Dy-EMF anions in acetone precipitated most of them, but [Dy_2_@C_80_]^−^ remained in solution (Fig. 2a and b). After filtration, acetone from the filtrate was evaporated, the residue was washed with acetonitrile to remove iodine and excess electrolyte salt, and the remaining [N(*n*-Bu)_4_][Dy_2_@C_80_] was then re-dissolved in acetone. Totally, around 1 mg of [N(*n*-Bu)_4_][Dy_2_@C_80_] required for its further study by AC magnetometry (see below) was accumulated. Its UV-vis spectrum is featureless and shows absorption to 800 nm, which is typical for M_2_@*I*_h_-C_80_ (Fig. 2e).^53,54^

The large difference in electron affinity and reduction potential between *I*_h_ and *D*_5h_ isomers of Dy_2_@C_80_ suggests that only [Dy_2_@*I*_h_-C_80_]^−^ remained in acetone solution after the reaction with iodine. However, since MS cannot distinguish the isomers, we analyzed the precipitate in more detail. Its mass spectrum appeared similar to that of the Dy-EMF anionic mixture in acetone before the reaction, except for the reduced signal of Dy_2_@C_80_ and increased Dy_2_@C_78_ (Fig. 2b). The presence of Dy_2_@C_80_ in both the precipitate and the acetone solution is the first indication of the separation of *I*_h_ and *D*_5h_ isomers. Moreover, the precipitate was found to be largely soluble in CS_2_ and toluene, which allowed its separation by HPLC and more thorough characterization (Fig. 2c). Dy@C_82_ was found in two fractions, while Dy_2_@C_78_ and Dy_2_@C_80_ each corresponded to a single HPLC fraction, whose Vis-NIR absorption spectra corresponded well to the literature data on M_2_@*D*_3h_-C_78_ and M_2_@*D*_5h_-C_80_ (Fig. 2e).^48,49,54^

We also analyzed whether the gradual addition of iodine can be used for step-wise selective oxidation and precipitation of individual Dy-EMF anions. HPLC traces of precipitates formed at subsequent I_2_-addition steps are compared in Fig. 2c. The oxidation potentials of Dy-EMF anions appeared too close to afford separation of individual compounds by a simple redox process, but the partial separation can be clearly seen. For this work, it is particularly important that [Dy_2_@*D*_5h_-C_80_]^−^ is oxidized at the very first steps, and the process is complete when some [Dy@C_82_]^−^ is still present in solution. It proves that [Dy_2_@*D*_5h_-C_80_]^−^ is fully precipitated by oxidation with an excess of I_2_ and that [Dy_2_@C_80_]^−^ remaining in the acetone solution is the sole *I*_h_ isomer.

The reaction of an acetone solution of Tb-EMF anions with iodine was similarly used to obtain [Tb_2_@*I*_h_-C_80_]^−^ (Fig. 2d) as well as neutral Tb_2_@C_78_ and Tb_2_@*D*_5h_-C_80_ (see their UV-vis-NIR spectra in Fig. 2e and further characterization details in the SI). However, it proved more difficult to separate [Tb_2_@*I*_h_-C_80_]^−^ from [Tb@C_82_]^−^ than for analogous Dy-EMFs. Therefore, after precipitation of Tb_2_@C_78_, Tb_2_@*D*_5h_-C_80_, Tb@C_80_ and some Tb@C_82_ by iodine, the remaining mixture of [Tb_2_@*I*_h_-C_80_]^−^ and [Tb@C_82_]^−^ was further separated by HPLC using a Poroshell column and acetone as an eluent (see the SI for further details).

To summarize, we developed a simple procedure for the isolation of M_2_@*I*_h_-C_80_ anions based on the redox extraction of the fullerene soot by DMF, followed by the oxidation and precipitation of the majority of EMFs by iodine, which leaves [M_2_@*I*_h_-C_80_]^−^ in solution. For M = Dy, the procedure is completely HPLC free, which is very remarkable given that HPLC separation is a common bottleneck in the synthesis of EMFs. To the best of our knowledge, this is the first HPLC-free isolation of EMF anions, while HPLC-free isolation was reported so far only for a handful of neutral EMFs.^55–60^ Further improvement of the procedure may be achieved by optimizing the solvent or solvent mixture instead of acetone, as some EMF anions were not fully dissolved by acetone. The procedure may also benefit from the more precise potential control that can be achieved, for instance, by electrolysis. Nonetheless, our main goal for this work, the isolation of [M_2_@*I*_h_-C_80_]^−^ for their magnetometry studies, was accomplished. The strategy we developed will also streamline the synthesis of the M_2_@*I*_h_-C_80_ derivatives, such as M_2_@C_80_(CF_3_) and M_2_@C_80_(CH_2_Ph), as the precipitation of other EMF anions before the derivatization will strongly simplify the separation of fullerene derivatives.

### Molecular structures and DFT calculations

The molecular structure of Dy_2_@*I*_h_(7)-C_80_(CF_3_) was established by single-crystal X-ray diffraction (SC-XRD). Single crystals were obtained by co-crystallization of the fullerene dissolved in toluene with nickel octaethylporphyrin (NiOEP) in benzene. X-ray diffraction data collection was carried out at the BESSY storage ring (BL14.2, Berlin-Adlershof, Germany).^61^ The XDSAPP2.0 suite was employed for data processing.^62,63^ The structure was solved by direct methods and refined using SHELXL-2018.^64^ The crystal data are presented in Table S2.

**Fig. 3:**
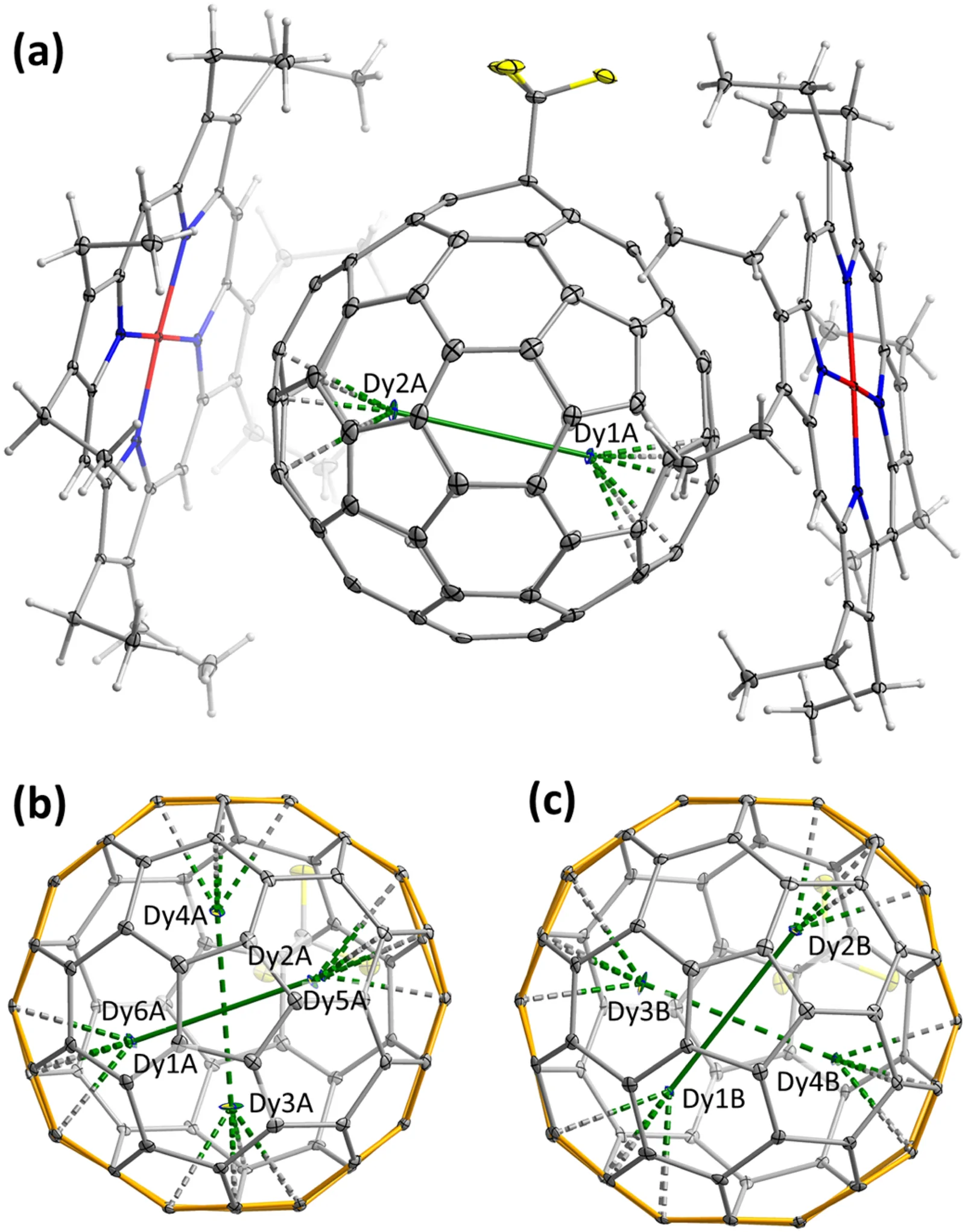
(a) A fragment of the single-crystal X-ray structure of 2Dy_2_@*I*_h_(7)-C_80_(CF_3_)·4NiOEP·1.58C_7_H_8_ 0.42C_6_H_6_ showing ordered fullerene molecule A with its major Dy sites (Dy1A and Dy2A, occupancies of 0.55) and two clamping NiOEP molecules. (b and c) Dy_2_@C_80_(CF_3_) molecules A (b, fully ordered) and B (c, only one of two overlapping molecules is shown) with all metal sites (occupancies 0.55, 0.19, and 0.26 for Dy1A/Dy2A, Dy3A/Dy4A, and Dy5A/Dy6A, respectively; 0.39 and 0.11 for Dy1B/Dy2B and Dy3B/Dy4B). The displacement parameters are shown at the 10% probability level. Color code: grey for carbon, green for Dy, yellow for F, blue for N, white for H, and red for Ni; the belt of hexagons around which the metal dimer tends to locate is highlighted dark yellow in (b) and (c).

The crystal structure of Dy_2_@*I*_h_(7)-C_80_(CF_3_) with an unusually large cell and three crystallographically different fullerene molecules closely resembles those of the recently reported Tb_2_@*I*_h_(7)-C_80_(CF_3_)^12^ and Nd_2_@*I*_h_(7)-C_80_(CF_3_).^11^ Each fullerene molecule is clamped by two NiOEP molecules, as shown in Fig. 3a. The ordered fullerene molecule (noted as site A) allows unambiguous structure determination as Dy_2_@*I*_h_(7)-C_80_(CF_3_), with the CF_3_ addition site at the 5/6/6 position as in other M_2_@C_80_(CF_3_) and M_2_@C_80_(CH_2_Ph) adducts. Four halves of the disordered fullerene molecules (sites B and C) are related by the *C*_2_ axis of the crystal passing through the molecule. Dy atoms coordinate fullerene cage hexagons. Dy_2_ shows disorder under the hexagonal belt of the fullerene cage, occupying three sites with occupancies 0.55/0.26/0.19 in molecule A and two sites in molecules B (0.38/0.12) and C (0.39/0.11). Due to the relatively high occupancies claimed by the main sites, the Dy–Dy bond length can still be reliably evaluated, giving 3.882(2) Å for Dy1A–Dy2A, 3.858(2) Å for Dy1B–Dy2B, and 3.847(3) Å for Dy1C–Dy2C.

The structures of [M_2_@*I*_h_-C_80_]^−^ anions (M = Gd, Tb, and Dy) were analysed based on DFT calculations at the PBE/def2-TZVPP level with a 4f-in-core effective potential for lanthanides. For comparison of M–M bond lengths in different cage environments, calculations were also performed for M_2_@C_79_N, M_2_@C_80_(CF_3_), and M_2_@C_80_(CH_2_Ph). In [M_2_@*I*_h_-C_80_]^−^, metal atoms coordinate two opposite cage hexagons in the *η*^6^-manner (Fig. 1). The *D*_2h_-symmetric structure with the central position of metal atoms above hexagons is a transition state with one imaginary frequency, while the true minimum has *C*_2h_ symmetry with a slight shift of metal atoms towards one of the 5/6 edges (Fig. 1); the energy difference is only 0.1 kJ mol^−1^. In M_2_@C_79_N, M_2_@C_80_(CF_3_), and M_2_@C_80_(CH_2_Ph), metals avoid N or C-sp^3^ sites, leading to a preferred location of the metal dimer around the belt of hexagons highlighted in Fig. 3b and c. Several such conformers span the energy range of only a few kJ mol^−1^, leading to a circular motion of the dimer at room temperature. Table 2 shows the data for the two lowest-energy structures, which coincide with experimental Dy1A/Dy2A and Dy5A/Dy6A positions in Dy_2_@C_80_(CF_3_)-A (Fig. 3). A small variation of the metal coordination in these two conformers leads to a variation of the M–M bond length by 0.05–0.06 Å. The metal-hexagon coordination in all derivatives is less central than in [M_2_@*I*_h_-C_80_]^−^. The M–M distances systematically shorten with the increase of the metal ionic radius from Dy to Gd (Table 2), suggesting that the geometry is controlled by the M–C bonding. The shortest M–M bonds are found in M_2_@C_79_N and bond lengths in M_2_@C_80_(CF_3_) and M_2_@C_80_(CH_2_Ph) are longer by ∼0.02 Å, while the longest M–M bonds are found in [M_2_@*I*_h_-C_80_]^−^.

**Table 2:** DFT-computed and experimental M–M bond lengths (M = Dy, Tb, Gd) and exchange coupling constants (M = Gd) in di-EMFs with single-electron M–M bonds and C_80_-*I*_h_ fullerene cages^a^

	*d*_Dy–Dy_, Å	*d*_Tb–Tb_, Å	*d*_Gd–Gd_, Å	*K*_Gd–e_, cm^−1^	*j*_Gd–Gd_, cm^−1^
[M_2_@C_80_]^−^-*C*_2h_	3.947	3.931	3.911	190.2	−1.4
M_2_@C_80_(CH_2_Ph)-1	3.909	3.894	3.883	193.0/187.9	−1.2
M_2_@C_80_(CH_2_Ph)-2	3.853	3.838	3.822	198.2/181.3	−0.8
Exp^b^	3.896(1)^10^			160 ± 10	
M_2_@C_80_(CF_3_)-1	3.910	3.895	3.878	193.2/187.4	−1.2
M_2_@C_80_(CF_3_)-2	3.853	3.840	3.824	198.5/181.2	−0.8
Exp^b^	3.882(2)	3.856(2)^12^	3.788(4)^13^		
M_2_@C_79_N-1	3.888	3.883	3.867	195.4/182.5	−1.1
M_2_@C_79_N-2	3.836	3.833	3.818	198.5/184.6	−1.1
Exp^b^	3.890(2)^16^	3.902(1)^2^	3.835(9)^4^	170 ± 10	

a Molecular structures are optimized at the PBE/def2-TZVPP level with a 4f-in-core ECP basis for lanthanides; exchange constants in Gd analogs are computed with broken-symmetry (BS) DFT at the PBE0-DKH/def2-TZVPP level with full-electron SARC-DKH basis sets for Gd. BS-DFT values were mapped on the spin Hamiltonian: 
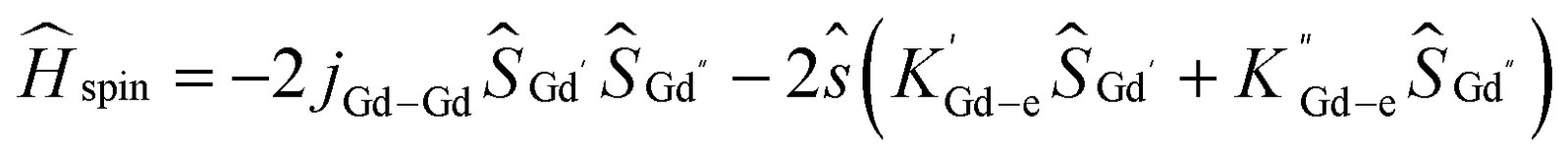
, see the SI for more details.

b Experimental bond lengths are SC-XRD values from this work for Dy_2_@C_80_(CF_3_) and from the literature for other molecules; only the values for the major metal sites are listed. Experimental exchange coupling constants are from ref. 9 for Gd_2_@C_80_(CH_2_Ph) and ref. 5 for Gd_2_@C_79_N.

### Magnetic properties

With the synthesis and isolation of Dy_2_@C_80_(CF_3_), [Dy_2_@C_80_]^−^ and [Tb_2_@C_80_]^−^, we can systematically study how magnetic properties of lanthanide dimers with 1e–2M bonds are affected by the modification of the host and analyze the difference between Dy and Tb in these settings. The analysis here will be performed for a set of eight di-EMFs with 1e–2M bonds, all based on the *I*_h_-C_80_ cage with a different degree of perturbation, from the pristine cage in [M_2_@C_80_]^−^, through addition of an exohedral group in M_2_@C_80_(CF_3_) and M_2_@C_80_(CH_2_Ph), to the substitution of one carbon atom with nitrogen in M_2_@C_79_N (Fig. 1).

**Fig. 4:**
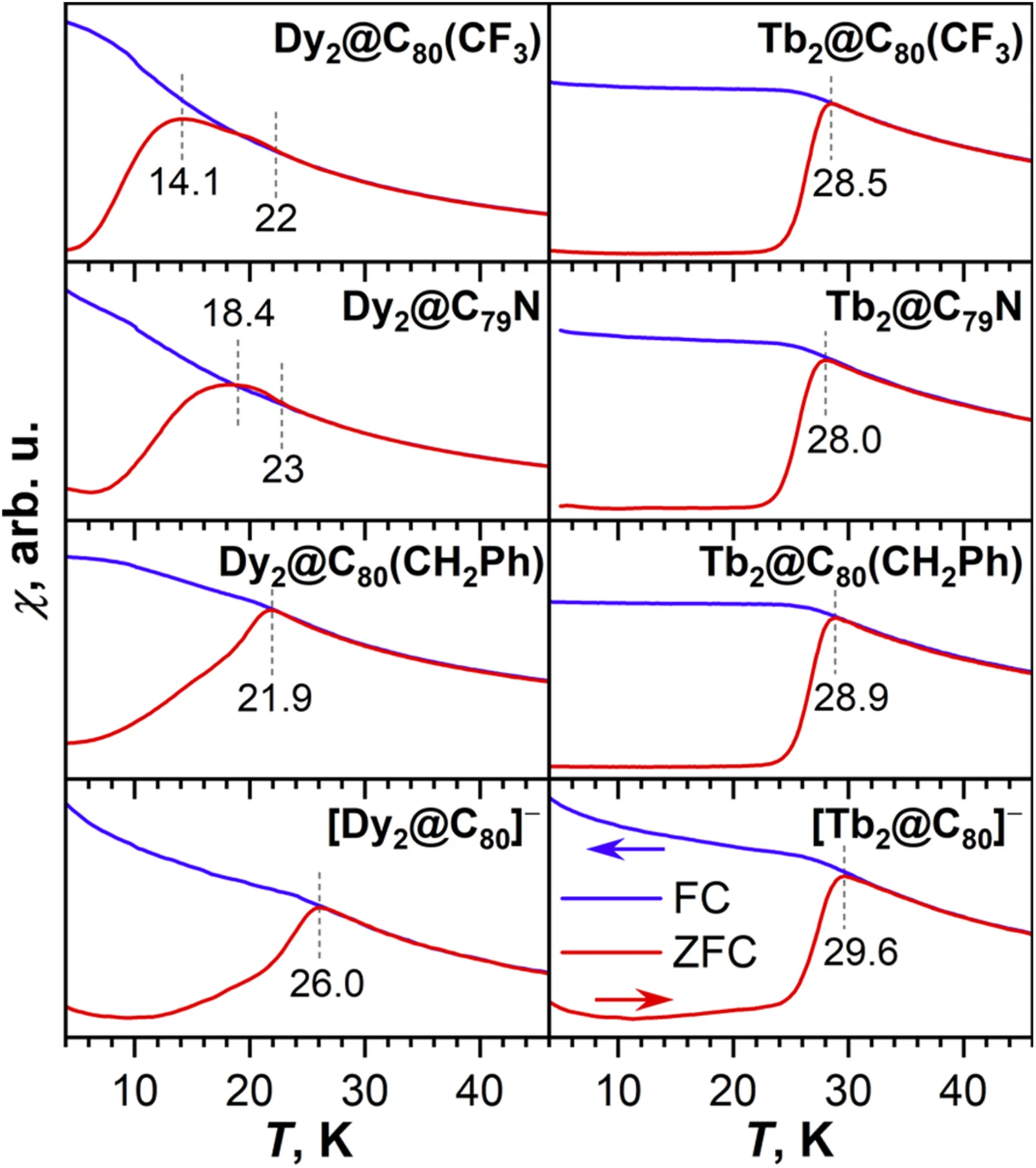
Determination of the blocking temperature of magnetization (*T*_B_ and *T*_irrev_) from the measurements of magnetic susceptibility during the in-field cooling (*χ*_FC_, blue curves) and warming of the zero-field-cooled sample (*χ*_ZFC_, red curves); sweep rate 5 K min^−1^ at 0.2 T. *T*_B_ is defined as the temperature of the *χ*_ZFC_ peak; *T*_irrev_ is defined as the temperature of the bifurcation point of *χ*_FC_ and *χ*_ZFC_, when it is different from the *χ*_ZFC_ peak.

**Fig. 5:**
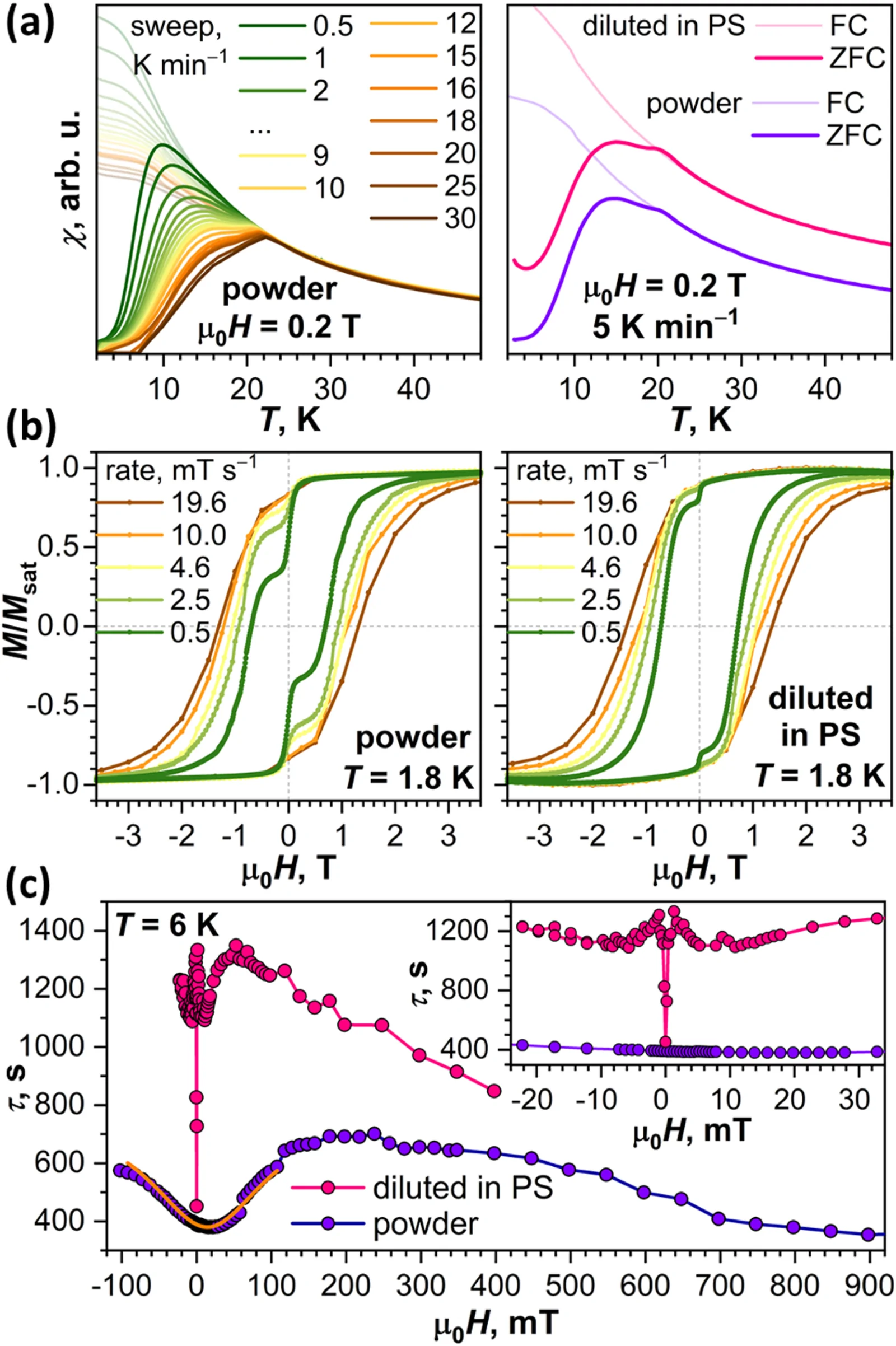
(a) *χ*_ZFC_ (full color) and *χ*_FC_ (pale) curves of Dy_2_@C_80_(CF_3_) measured at 0.2 T with different temperature sweep rates (left); comparison of *χ*_ZFC_ and *χ*_FC_ curves for powder and diluted Dy_2_@C_80_(CF_3_) measured at 0.2 T with a sweep rate of 5 K min^−1^ (right). (b) Magnetic hysteresis loops of Dy_2_@C_80_(CF_3_) powder (left) and Dy_2_@C_80_(CF_3_) diluted in polystyrene (PS, right), measured at 1.8 K with different sweep rates; for the PS-diluted sample, the linear part of a strong diamagnetic background is subtracted for the sake of a better comparison. (c) Magnetization relaxation times of powder and diluted Dy_2_@C_80_(CF_3_) measured at 6 K in different magnetic fields after the fast field sweep from 7 T; orange curve is the Lorentzian function fitting the distribution near zero field; inset shows the low-field range with the sharp QTM resonance of the diluted sample; the field scale is corrected by 2.2 mT, the residual magnetization of the superconducting magnet after the field ramp from 7 T to 0 T.

**Fig. 6:**
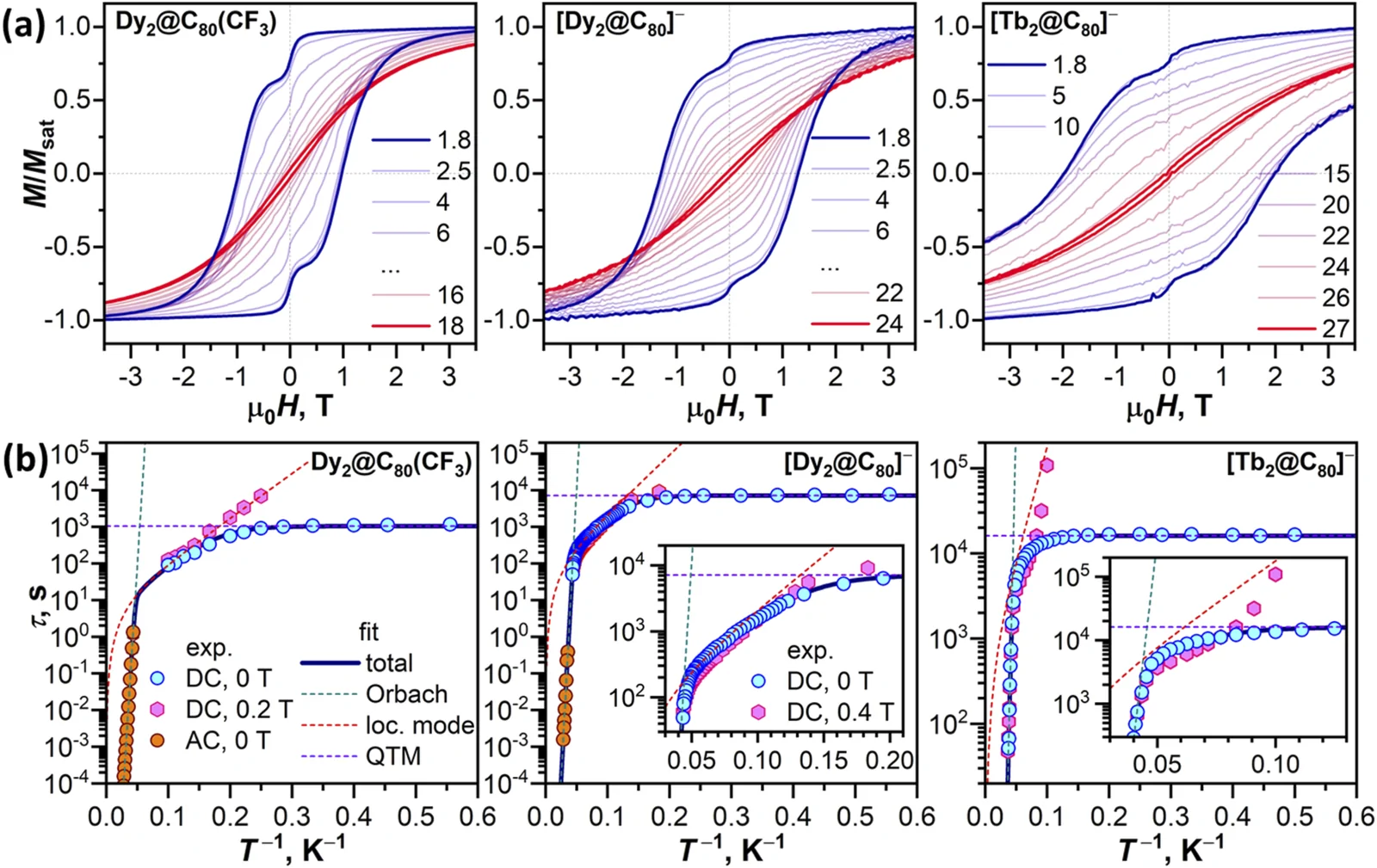
(a) Magnetic hysteresis measured at different temperatures for Dy_2_@C_80_(CF_3_), [Dy_2_@C_80_]^−^, and [Tb_2_@C_80_]^−^; average sweep rate: 2.9 mT s^−1^. (b) Relaxation times of Dy_2_@C_80_(CF_3_), [Dy_2_@C_80_]^−^, and [Tb_2_@C_80_]^−^ and their fitting with the model, which includes the Orbach, local mode, and QTM mechanisms; contributions of individual processes are plotted with dashed lines.

**Fig. 7:**
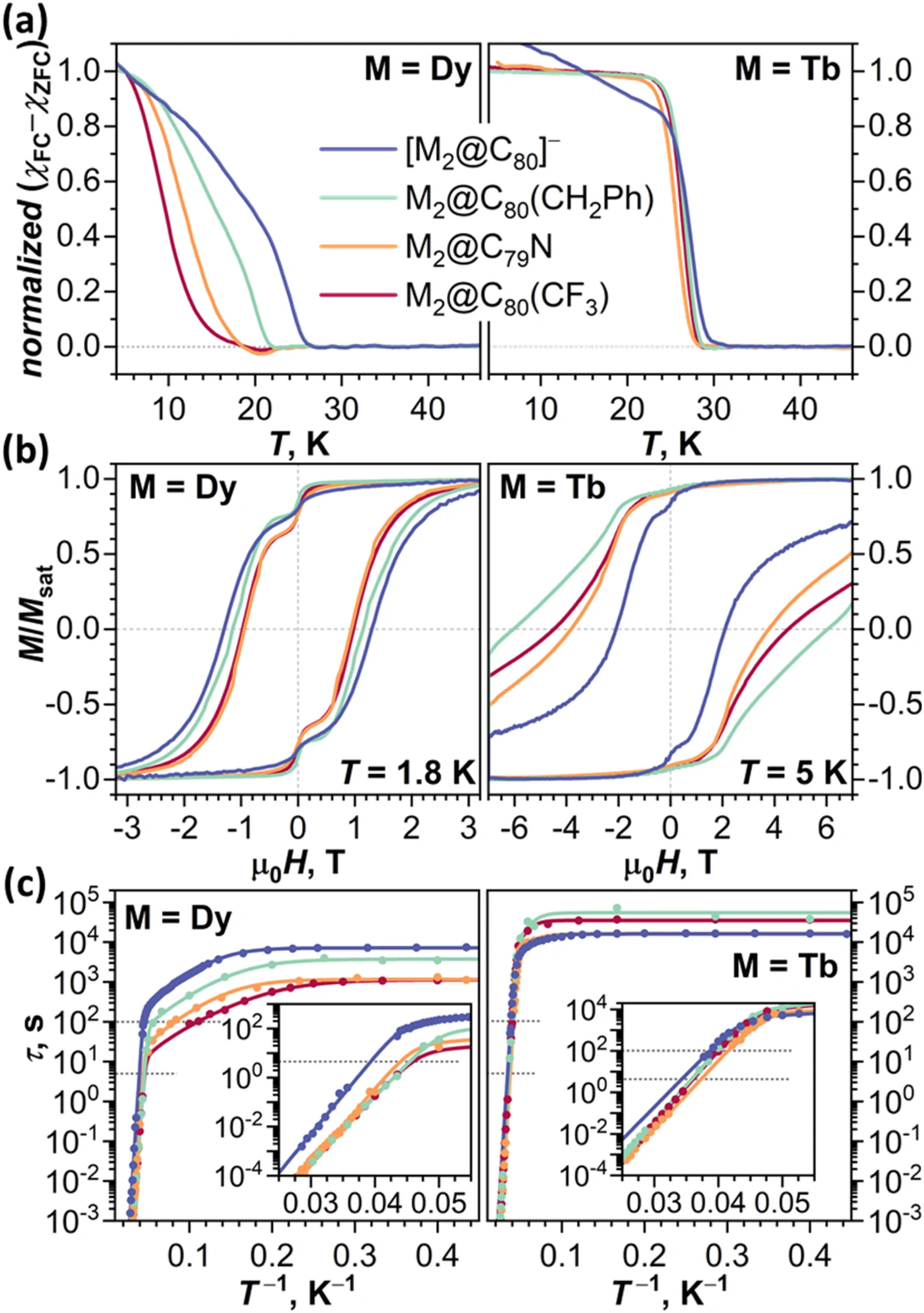
Comparison of the SMM behavior of Dy and Tb dimetallofullerenes. (a) Difference between *χ*_FC_ and *χ*_FC_ (sweep rate: 5 K min^−1^ at 0.2 T; for the sake of comparison, the curves are normalized to the 5 K value for Dy and the 15 K value for Tb). (b) Magnetic hysteresis curves measured at 1.8 K (Dy) and 5 K (Tb) and an average sweep rate of 2.9 mT s^−1^; note the different field scales for Dy and Tb. (c) Zero-field magnetization relaxation times and fitted temperature dependencies in Arrhenius coordinates; insets show expansions of the low-temperature ranges; horizontal dotted lines are plotted at 100 s and 5 s, corresponding to *T*_B100_ and *T*_B_/*T*_irrev_, respectively.

From the magnetism point of view, the 1e–2M bond in lanthanide di-EMFs is described as a three-center [M^3+^–e–M^3+^] system, whose effective spin Hamiltonian can be presented in the following form:

1 *Ĥ*_spin_ = *Ĥ*_LF_′ + *Ĥ*_LF_″ − 2*K*^eff^*ŝ*(*Ĵ*_M_′ + *Ĵ*_M_″)

where *Ĥ*_LF_ is a single-ion ligand-field Hamiltonian, *ŝ* is the spin momentum of the unpaired valence electron (*S* = 1/2), and *K*^eff^ is the exchange coupling constant between the unpaired valence electron and the 4f-shell of lanthanide ions.^1,5,9,10^ Lanthanide ions are considered in their ground-state M^3+^ multiplet with the corresponding angular momentum operator *Ĵ*_M_. In di-EMFs with 1e–2M bonds, Tb and Dy attain a moderately strong uniaxial single-ion magnetic anisotropy with *J*_z_ values of ±6 and ±15/2 respectively, and with the quantization axis aligned along the M–M bond, although some degree of non-linearity is possible.^1^ In the ground magnetic state of the [M^3+^–e–M^3+^] dimer, all moments are coupled ferromagnetically, as discussed in more detail and studied theoretically elsewhere.^18,65–67^ The static magnetic properties of all di-EMFs in Dy_2_ and Tb_2_ series are thus quite similar, but they do show considerable differences in the dynamics of (de)magnetization. Therefore, in the following, we focus on their SMM characteristics, including the blocking temperature of magnetization (*T*_B_), magnetic hysteresis, and magnetization relaxation times (Fig. 4–6). Fig. 7 summarizes SMM data for both Dy_2_ and Tb_2_ series.

#### Blocking temperature of magnetization *T*_B_

A classical definition of *T*_B_ is based on the magnetization temperature dependencies measured for field-cooled (FC) and zero-field-cooled (ZFC) samples.^68^ ZFC/FC measurements for Dy and Tb dimetallofullerene series are compared in Fig. 4. Most of them show the standard behavior with the peak of the ZFC curve (defined as *T*_B_) coinciding with the bifurcation point between ZFC and FC. However, ZFC peaks of Dy_2_@C_80_(CF_3_) and Dy_2_@C_79_N occur at lower temperatures than ZFC/FC bifurcation points, the latter being defined as *T*_irrev_. *T*_B_ of Dy_2_@C_80_(CF_3_) at 14.1 K and of Dy_2_@C_79_N at 18.4 K are noticeably lower than *T*_B_ of Dy_2_@C_80_(CH_2_Ph) at 21.9 K, but *T*_irrev_ values for all of them are close to 22–23 K. [Dy_2_@C_80_]^−^ shows a considerable increase of *T*_B_ to 26 K, demonstrating that the lack of the cage defect is instrumental for slowing the relaxation of magnetization. In the Tb_2_ series, all compounds show classical ZFC/FC behavior with *T*_B_ values between 28 and 30 K. With a *T*_B_ of 29.6 K, [Tb_2_@C_80_]^−^ has the highest value in the series.

The unusual double-peak shape of the ZFC curve of Dy_2_@C_80_(CF_3_) and a considerable difference between *T*_B_ and *T*_irrev_ prompted us to perform ZFC/FC measurements at different sweep rates from 0.5 to 30 K min^−1^ (Fig. 5a). At a slow rate of 0.5 K min^−1^, the first peak occurs at 10 K with a high intensity. With the increase of the sweep rate, the peak shifts to higher temperature, reaching 15.4 K at 10 K min^−1^, but then retains its position and simultaneously decreases in intensity at faster sweep rates. Above 20 K min^−1^, it is no longer clearly visible. The second peak becomes observable at sweep rates above 3 K min^−1^ and shows a modest rate dependence, from 20 K at 3 K min^−1^ to 22.3 K at 30 K min^−1^. As the first peak gradually decreases in intensity, the shape of the ZFC curve transforms to a more common one with a single maximum at the ZFC/FC bifurcation point. This evolution suggests that the two peaks in the ZFC curve are caused by contributions of different relaxation mechanisms (*vide infra*). Note that the appearance of two peaks in ZFC curves has recently been reported for some Er-SMMs and similarly associated with two relaxation mechanisms.^69^

#### Magnetic hysteresis

Below the blocking temperature, SMMs develop a hysteresis in the field dependence of magnetization. Fig. 6a shows magnetic hysteresis curves measured for Dy_2_@C_80_(CF_3_), [Dy_2_@C_80_]^−^, and [Tb_2_@C_80_]^−^ with an average sweep rate of 2.9 mT s^−1^. The hysteresis in Dy_2_@C_80_(CF_3_) is observed up to 18 K. The loops have a characteristic step at zero field corresponding to the quantum tunneling of magnetization (QTM). The coercive field *H*_c_ is 0.97 T at 1.8 K and almost doubles when the sweep rate is varied 40-fold, from *H*_c_ = 0.70 T at 0.5 mT s^−1^ to *H*_c_ = 1.31 T at 20 mT s^−1^.

The hysteresis exhibited by [Dy_2_@C_80_]^−^ is broader (*H*_c_ = 1.30 T at 1.8 K) and the loops remain open up to 24–25 K. The QTM step at zero field is visible but considerably smaller than in Dy_2_@C_80_(CF_3_). The hysteresis shape of Dy_2_@C_79_N measured under the same conditions is very similar to that of Dy_2_@C_80_(CF_3_), while Dy_2_@C_80_(CH_2_Ph) has an intermediate *H*_c_ of 1.13 T (Table 3 and Fig. 7b). Dy_2_@C_80_(CF_3_) and [Dy_2_@C_80_]^−^ are thus the softest and the hardest SMMs in the Dy_2_ series, respectively.

**Table 3:** SMM parameters of Dy and Tb dimetallofullerenes a^,^^b^

EMF	*T*_B_/*T*_irrev_ K	*T*_B100_ K	*H*_c_ T	*τ*_QTM_ h	*τ*_Orb_ s 10^−12^	*U* ^eff^ K	*τ*_loc_ s	Δ_loc_ K
Dy_2_@C_80_(CF_3_)	14.1/22	9.0	0.97	0.29(1)	3.4(5)	616(4)	5.9(6)	28(1)
Dy_2_@C_79_N	18.4/23	12.1	0.94	0.32(4)	0.3(1)	695(12)	9(5)	32(6)
Dy_2_@C_80_(CH_2_Ph)	21.9	18.2	1.15	0.97(7)	2.5(9)	623(13)	18(2)	37(1)
[Dy_2_@C_80_]^−^	26.0	22.3	1.30	1.99(8)	1.5(4)	730(9)	50(5)	38(1)
Tb_2_@C_80_(CF_3_)	28.5	24.9	4.49 c	9.8(6)	1.3(2)	800(5)		
Tb_2_@C_79_N	28.0	24.1	3.82	4.5(3)	2.4(3)	756(4)		
Tb_2_@C_80_(CH_2_Ph)	28.9	25.2	6.04 d	15(2)	1.4(3)	802(7)		
[Tb_2_@C_80_]^−^	29.6	25.9	2.10	4.6(1)	78(99)	721(32)		
Tb_2_@C_80_(CH_2_Ph)-pyr_2_	30.3	26.6	5.42	16.9(3)	321(57)	709(5)		

a *T*_B_/*T*_irrev_ are determined at a sweep rate of 5 K min^−1^ in a field of 0.2 T and *H*_c_ is determined at a sweep rate of 2.9 mT s^−1^ at a temperature of 1.8 K for Dy di-EMFs and 5 K for Tb di-EMFs.

b Relaxation times of Dy_2_@C_80_(CF_3_), [Dy_2_@C_80_]^−^, and [Tb_2_@C_80_]^−^ used in the fits are from this work; relaxation times of other compounds are from ref. 16 for Dy_2_@C_79_N, ref. 10 for Dy_2_@C_80_(CH_2_Ph), ref. 12 for Tb_2_@C_80_(CF_3_), ref. 1 for Tb_2_@C_79_N, ref. 9 for Tb_2_@C_80_(CH_2_Ph), and ref. 27 for Tb_2_@C_80_(CH_2_Ph)-pyr_2_.

c For Tb_2_@C_80_(CF_3_), *H*_c_ at 1.8 K changes from 2.28 T at 0.6 mT s^−1^ to 4.51 T at 2.9 mT s^−1^ and to more than 7 T at 24 mT s^−1^.^12^

d For Tb_2_@C_80_(CH_2_Ph), *H*_c_ at 5 K increases to 8.21 T at a sweep rate of 10 mT s^−1^.^9^

The hysteresis of [Tb_2_@C_80_]^−^ remains open up to 27 K (Fig. 6a). Its shape shows that the higher *T*_B_ does not necessarily correspond to the broader hysteresis. The coercive field of [Tb_2_@C_80_]^−^, *H*_c_ = 2.10 T at 5 K, is the smallest in the whole Tb_2_ series (Table 3 and Fig. 7b), while still being considerably larger than for [Dy_2_@C_80_]^−^. The broadest hysteresis among Tb_2_ di-EMFs at a sweep rate of 2.9 mT s^−1^ is observed for Tb_2_@C_80_(CH_2_Ph) (*H*_c_ = 6.04 T) and its pyrrolidine derivative (5.42 T), followed by Tb_2_@C_80_(CF_3_) with 4.49 T and Tb_2_@C_79_N with 3.82 T.

#### Magnetization relaxation times

The most comprehensive characterization of the SMM behavior is provided by the temperature dependence of the magnetization relaxation times (*τ*). Relaxation times were determined using the DC technique, which allows measurements of *τ* longer than 100 s, but becomes increasingly unreliable for shorter times. Short relaxation times (*τ* < 1 s) of Dy_2_@C_80_(CF_3_) and [Dy_2_@C_80_]^−^ were studied by the AC technique. The lower yield and smaller isolated amount of [Tb_2_@C_80_]^−^ did not allow its study by the AC method. The measured temperature dependencies of relaxation times are plotted in Fig. 6b.

Spin-lattice relaxation of Dy_2_@C_80_(CF_3_) shows three temperature regimes. Below 4 K, zero-field relaxation times are temperature independent, which is the characteristic feature of the QTM. Between 4 K and 14 K, a new process with a modest temperature acceleration sets in and becomes progressively more visible. Above 23 K, *τ* follows a linear Arrhenius regime, which is characteristic of the Orbach relaxation mechanism. The transition between the second and the third regimes falls into the range, in which neither DC nor AC can enable reliable determination of relaxation times. Nonetheless, the whole temperature dependence can be fitted by a combination of three mechanisms:

2 *τ*^−1^(*T*) = τ_QTM_^−1^ + τ_Orb_^−1^exp(−*U*^eff^/*T*) + τ_loc_^−1^exp(Δ_loc_/*T*){exp(Δ_loc_/*T*) − 1}^−2^

The first term corresponds to the QTM, the second is the Orbach mechanism with the attempt time *τ*_Orb_ and the energy barrier *U*^eff^, while the third term describes the relaxation driven by a local vibrational mode with the frequency *Δ*_loc_ and attempt time *τ*_loc_. For Dy_2_@C_80_(CF_3_), the fit was performed simultaneously for zero-field and in-field relaxation times (the latter without the QTM term) and gave a *τ*_QTM_ of 17 minutes, a high-temperature Orbach regime with *τ*_Orb_ = 3.4 × 10^−12^ s^−1^ and *U*^eff^ = 616 K, and the local mode with a *τ*_loc_ of 6 s and a *Δ*_loc_ of 28 K (see Table 3).

Three relaxation regimes are also identified for [Dy_2_@C_80_]^−^. However, the QTM with a *τ*_QTM_ of 2 hours is considerably slower, and the Orbach mechanism is characterized by a shorter attempt time but a higher barrier, *τ*_Orb_ = 1.5 × 10^−12^ s^−1^ and *U*^eff^ = 730 K. The local-mode regime and its transition to the Orbach regime now fall into the DC-accessible range, and we performed the measurements with small temperature steps to ensure a comprehensive description of the relaxation in this interval (Fig. 6b). In comparison to Dy_2_@C_80_(CF_3_), the *τ*_loc_ of [Dy_2_@C_80_]^−^ is much longer, 50 s, and the *Δ*_loc_ of 38 K is likewise higher.

The earlier studied Dy_2_@C_80_(CH_2_Ph)^10^ and Dy_2_@C_79_N^16^ exhibited a similar sequence of three relaxation regimes. For consistency, we also fitted their temperature dependencies with eqn (2). The QTM time of Dy_2_@C_79_N is close to that of Dy_2_@C_80_(CF_3_), while the *τ*_QTM_ of Dy_2_@C_80_(CH_2_Ph) is near 1 h. The local mode energies are 32 K for Dy_2_@C_79_N and 37 K for Dy_2_@C_80_(CH_2_Ph), while their attempt times are 9 and 18 s, respectively. Interestingly, the parameters of the Orbach mechanism with *U*^eff^ near 620 K are very close for Dy_2_@C_80_(CH_2_Ph) and Dy_2_@C_80_(CF_3_), whereas the barriers of Dy_2_@C_79_N (695 K) and [Dy_2_@C_80_]^−^ (730 K) are noticeably higher.

The magnetization relaxation times of [Tb_2_@C_80_]^−^ show a two-segment temperature dependence (Fig. 6b), similar to what was observed in other Tb di-EMFs (Fig. 7c). The low-temperature part below 10–15 K is dominated by the QTM with a *τ*_QTM_ of 4.1 hours, which at higher temperature switches to the Orbach regime with *τ*_Orb_ = 8 × 10^−11^ s^−1^ and *U*^eff^ = 721 K. The transition between the two regimes is not very sharp and may still involve another relaxation mechanism, but its parameters cannot be determined reliably and will not be discussed. When compared to other Tb di-EMFs, the *τ*_QTM_ of [Tb_2_@C_80_]^−^ is one of the shortest on a par with Tb_2_@C_79_N^1^ (Table 3). The longest QTM times of 15–17 h are found for Tb_2_@C_80_(CH_2_Ph)^9^ and its pyrrolidine cycloadduct Tb_2_@C_80_(CH_2_Ph)-pyr_2_,^27^ while Tb_2_@C_80_(CF_3_) has an intermediate *τ*_QTM_ value of 9.8 h.^12^ The Orbach barrier of [Tb_2_@C_80_]^−^, *U*^eff^ = 720 K, is comparably low *versus* 800 K in Tb_2_@C_80_(CF_3_) and Tb_2_@C_80_(CH_2_Ph) and 756 K in Tb_2_@C_79_N.

#### QTM and dilution

All studied Dy and Tb di-EMFs exhibit the low-temperature QTM regime, and the variation of *τ*_QTM_ values is one of the most apparent differences between these compounds. The QTM for a di-EMF with a 1e–2M bond involves a simultaneous flip of the whole coupled magnetic moment of two metal atoms and the unpaired valence spin. At least two main factors affecting the QTM can be pointed out. One is the transition probability, which is a function of the local anisotropy. Evaluation of this factor with a purely experimental approach is hardly possible and will require detailed computational modelling of single-ion anisotropy and exchange coupling. Another well-known factor is the intermolecular dipolar interactions, which produce local dipolar fields (*B*^dip^) experienced by SMM molecules in a powder or crystal.^69–80^ Transversal *B*^dip^_⊥_ is believed to be responsible for opening the tunneling gap in Kramers systems, while longitudinal *B*^dip^_‖_ determines the magnetic field range around the level crossing, in which the QTM still can happen. To analyze the role of intermolecular interactions, we studied the field dependence of relaxation time and performed magnetic dilution.

Selected measurements of the relaxation times of di-EMFs in external fields of 0.2–0.4 T demonstrate their drastic elongation in comparison to zero-field. This is caused by the quenching of the QTM mechanism when the energy levels of the ground-state doublet are split by the Zeeman effect and go out of resonance. However, when the external field is smaller than the largest possible longitudinal dipolar field inside the sample, the internal and external fields can compensate each other, still making the QTM possible. To evaluate the distribution of *B*^dip^, we studied the field dependence of relaxation times. Di-EMFs have very slow relaxation at low temperatures, especially when the QTM is quenched, which makes the detailed study very time consuming. Dy_2_@C_80_(CF_3_) was chosen for a case study, as it has relatively short *τ*_QTM_, and the measurements were performed at 6 K, when the thermal mechanism is already relatively fast, but the QTM contribution is still strong. As common for systems with zero-field QTM, the fastest relaxation of Dy_2_@C_80_(CF_3_) is found near zero field (Fig. 5c). As the field is applied, *τ* first increases, reaches its maximum values at 0.2–0.3 T, and decreases again at higher fields due to the direct mechanism. Importantly, the shape of the *τ*(H) dependence near zero field is well described by the Lorentzian peak centered at 17 ± 1 mT with a full width at half maximum of 190 ± 14 mT. The Lorentzian shape of the field dependence of *τ*_QTM_ was suggested by Villain *et al.*^81,82^ and was later popularized in the two-parameter form, τ_QTM_^−1^(H) = *B*_1_(1 + *B*_2_*H*^2^)^−1^.^83^ The distribution determined for Dy_2_@C_80_(CF_3_) is quite broad, which is understandable given the large magnetic moment and compact size of fullerene molecules. Typical average *B*^dip^ in Dy-SMMs is in the range of a few to tens of mT,^73,76,80,84,85^ and the value assumed in the modelling of QTM in Dy-SMMs is 20 mT.^78,86^ The shift of the peak center from zero field in Fig. 5c is also quite remarkable. In a non-magnetized sample, the dipolar field distribution is symmetric and centered at zero, but it becomes asymmetric once the sample attains a certain magnetization.^76^ Thus, during the relaxation process, when more and more magnetic moments flip, the distribution changes continuously. Therefore, the relaxation time determined from the magnetization decay represents an average situation and should reflect the asymmetry of the dipolar field distribution in the partially magnetized sample.

To minimize the effect of internal dipolar fields, Dy_2_@C_80_(CF_3_) was diluted in polystyrene by mixing toluene solutions of fullerene and polymer and then allowing the solvent to evaporate. Dilution did not affect the blocking temperature (Fig. 5a), but strongly suppressed the QTM step in hysteresis curves (Fig. 5b). The field dependence of relaxation time in the diluted sample also changed spectacularly (Fig. 5c). The QTM resonance is observed at zero field and is very sharp, with a width of less than 1 mT, and we had to measure the field dependence with a step of only 0.2 mT not to miss it. Given the strong influence of intermolecular interactions on the QTM, the factors controlling the distance between fullerene molecules, including the presence and the size of exohedral groups, may play a certain role. However, the variation of *τ*_QTM_ values is different in Dy_2_ and Tb_2_ series and does not seem to correlate with the type of derivatization.

#### Local mode and *T*_B_

All Dy di-EMFs consistently show the presence of an intermediate regime, interpreted as a local vibrational mode contribution. The *Δ*_loc_ values are all below 40 K (27 cm^−1^), which corresponds to the frequency range of lateral metal modes, in which metal atoms oscillate parallel to the fullerene surface. In di-EMFs, these modes have a translational and librational character of the metal dimer. By virtue of their low frequencies and facile mixing with lattice modes, these vibrations should have an enhanced contribution to the spin-lattice relaxation.^87–91^ The importance of lateral modes for relaxation was also pointed out in the pulsed-EPR studies of Eu^II^@C_2*n*_ EMFs^30^ and Y_2_@C_80_(CH_2_Ph).^17^ CF_3_ and CH_2_Ph adducts additionally have some low-frequency addend-based vibrations, but such modes do not seem to play a strong role;^17^ otherwise, the local-mode regime in Dy_2_@C_80_(CF_3_) and Dy_2_@C_80_(CH_2_Ph) would be more pronounced than in [Dy_2_@C_80_]^−^ and Dy_2_@C_79_N.

The local mode mechanism is responsible for the considerable variation between *T*_B_ and *T*_B100_ values in the Dy_2_ series and the double-peak shape of ZFC curves for some of them. The *τ* of 100 s falls into the range of the local mode regime for Dy_2_@C_80_(CF_3_) and Dy_2_@C_79_N, the transition range between the local mode and Orbach regimes for Dy_2_@C_80_(CH_2_Ph), and the Orbach regime for [Dy_2_@C_80_]^−^. Local mode parameters vary considerably along the Dy_2_ series, and so do the *T*_B100_ values. At the temperatures of the low-*T* peaks in the ZFC curves of Dy_2_@C_80_(CF_3_) and Dy_2_@C_79_N, *τ* is some tens of seconds long and is still determined by the local-mode mechanism. However, fast temperature sweep rates can outpace this relaxation process, which leads to the gradual disappearance of the low-*T* peak at sufficiently fast sweeps as observed for Dy_2_@C_80_(CF_3_) (Fig. 5a). In the temperature range of the higher-*T* ZFC peaks (or the only ZFC peaks for Dy_2_@C_80_(CH_2_Ph) and [Dy_2_@C_80_]^−^), the relaxation is determined solely by the Orbach mechanism, and the *T*_irrev_ values of Dy_2_@C_80_(CF_3_) and Dy_2_@C_79_N are very close to the *T*_B_ of Dy_2_@C_80_(CH_2_Ph). [Dy_2_@C_80_]^−^ remains an outlier with higher *T*_B_ because its Orbach relaxation is also slower. In the Tb_2_ series, the local mode mechanism is not pronounced, and the transition between the QTM and Orbach regimes happens at *τ* longer than 10^4^ s. At shorter times, the relaxation is determined only by the Orbach mechanism (Fig. 7c), leading to similar *T*_B100_ and *T*_B_ values for all Tb di-EMFs. In this situation, further functionalization of the cage may even slightly increase the blocking temperature despite the symmetry lowering, as was found for the Tb_2_@C_80_(CH_2_Ph)-pyr_2_ adduct, obtained by pyrrolidine addition to Tb_2_@C_80_(CH_2_Ph).^27^ More extended derivatization of the fullerene cage may also affect the potential energy surface for the metal motion inside the cage and thereby the efficiency of the local mode mechanism in the Dy series. It remains to be seen if deeper functionalization can also increase the blocking temperature of Dy-based di-EMF.

#### Relaxation barrier and exchange interactions

Both Dy_2_ and Tb_2_ series show visible variations of *U*^eff^ values. The relaxation barrier in di-EMFs with 1e–2M bonds is associated with the exchange-excited state, in which the 4f-magnetic moment of one of the metal ions is flipped.^9,10^ Under Hamiltonian (1), the energy of this exchange-excited state should scale with *K*^eff^, but also reflect the variation of single-ion anisotropy. The theoretical study of Gd_2_@C_2*n*_ fullerenes by Rajaraman *et al.* revealed a strong dependence of exchange parameters on the fullerene size and the Gd–Gd distance.^67^ However, our calculations of *K*_Gd–e_ and *j*_Gd–Gd_ constants for the Gd_2_ series (Table 2) show that the variation of the Gd–Gd bond length with modification of the C_80_ cage is not sufficient to induce substantial changes in exchange parameters. We therefore suggest that the variation of magnetic anisotropy in the series should be a more important factor. Besides, the contribution of higher multipolar terms in the exchange^19^ and involvement of several excited states in the Orbach mechanism (which makes *U*^eff^ a weighted average of excited state energies^78,92^) can also affect the barrier.

#### Single-ion anisotropy

**Fig. 8:**
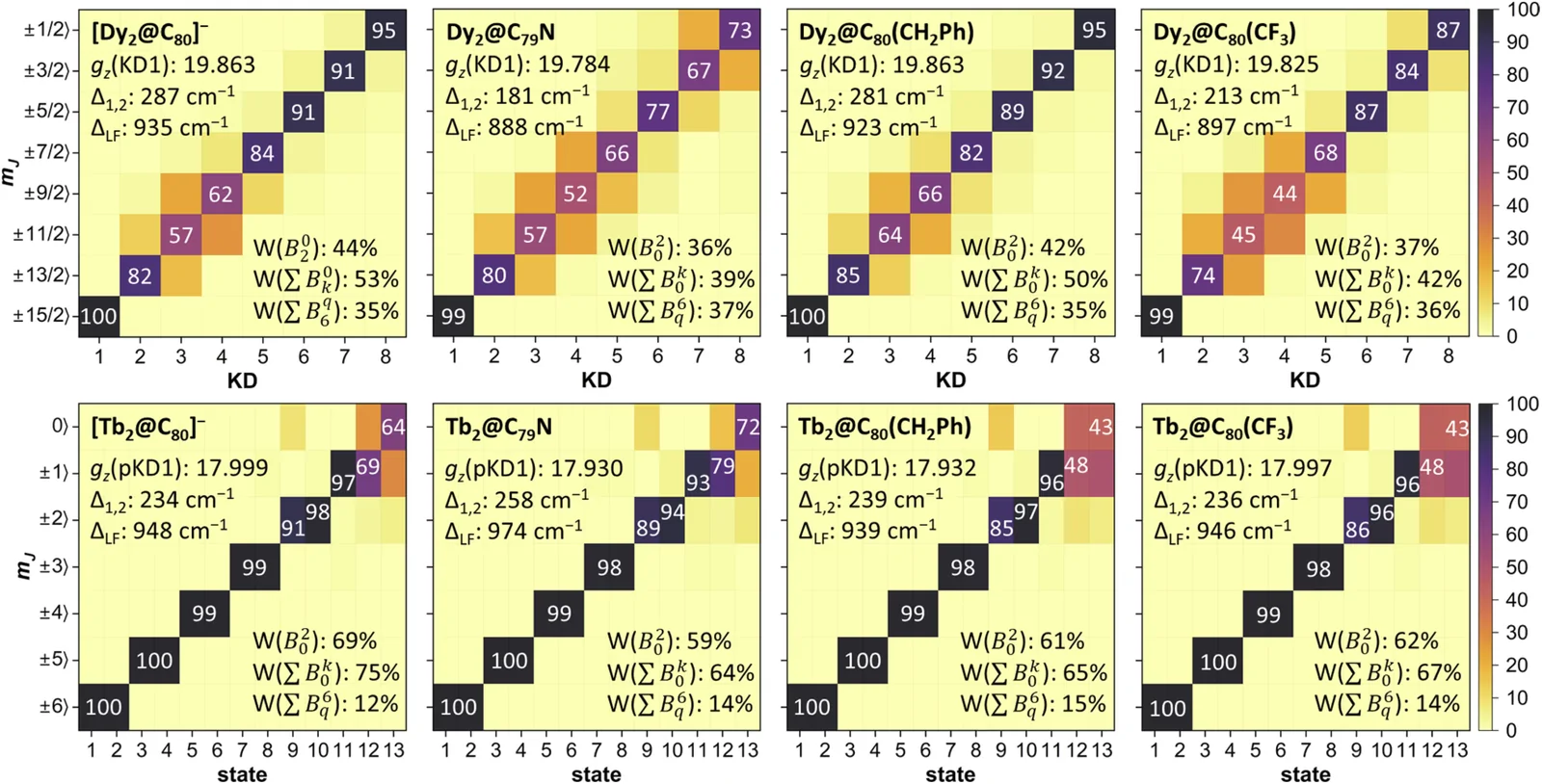
Representation of the ligand-field states of Dy (upper row) and Tb ions (bottom row) in the *m*_J_ basis (in %, the contribution of the leading *m*_J_ term is listed for each state in white font). Also listed for each compound are the pseudospin *g*_z_ value of the lowest-energy Kramers doublet (pseudo-doublet for Tb), the splitting between the ground state and the first excited doublet (Δ_1,2_), the total ligand-field (LF) splitting of the ground-state multiplet (Δ_LF_), the weight (W) of the *B*_0_^2^ ligand-field parameter in the LF splitting, and the net weights of axial terms (∑*B*_0_^k^) and hexacontadecapolar terms (∑*B*_q_^6^).

To evaluate the influence of the chemical environment on the single-ion magnetic anisotropy and its possible role in relaxation dynamics of Dy and Tb di-EMFs, we performed CASSCF/RASSI-SO calculations. We note that it is not yet possible to use a complete *ab initio* description of the metal dimer with the 1e–2M bond, and we only computed single lanthanide centers, while replacing the second center with Y and quenching the unpaired electron on the M–M bonding orbital with an additional electron. Ligand-field (LF) splitting values for Dy and Tb ions are listed in the SI (Table S10), while Fig. 8 illustrates the composition of LF states in the *m*_J_ basis and lists selected numerical parameters used in the discussion.

**Fig. 9:**
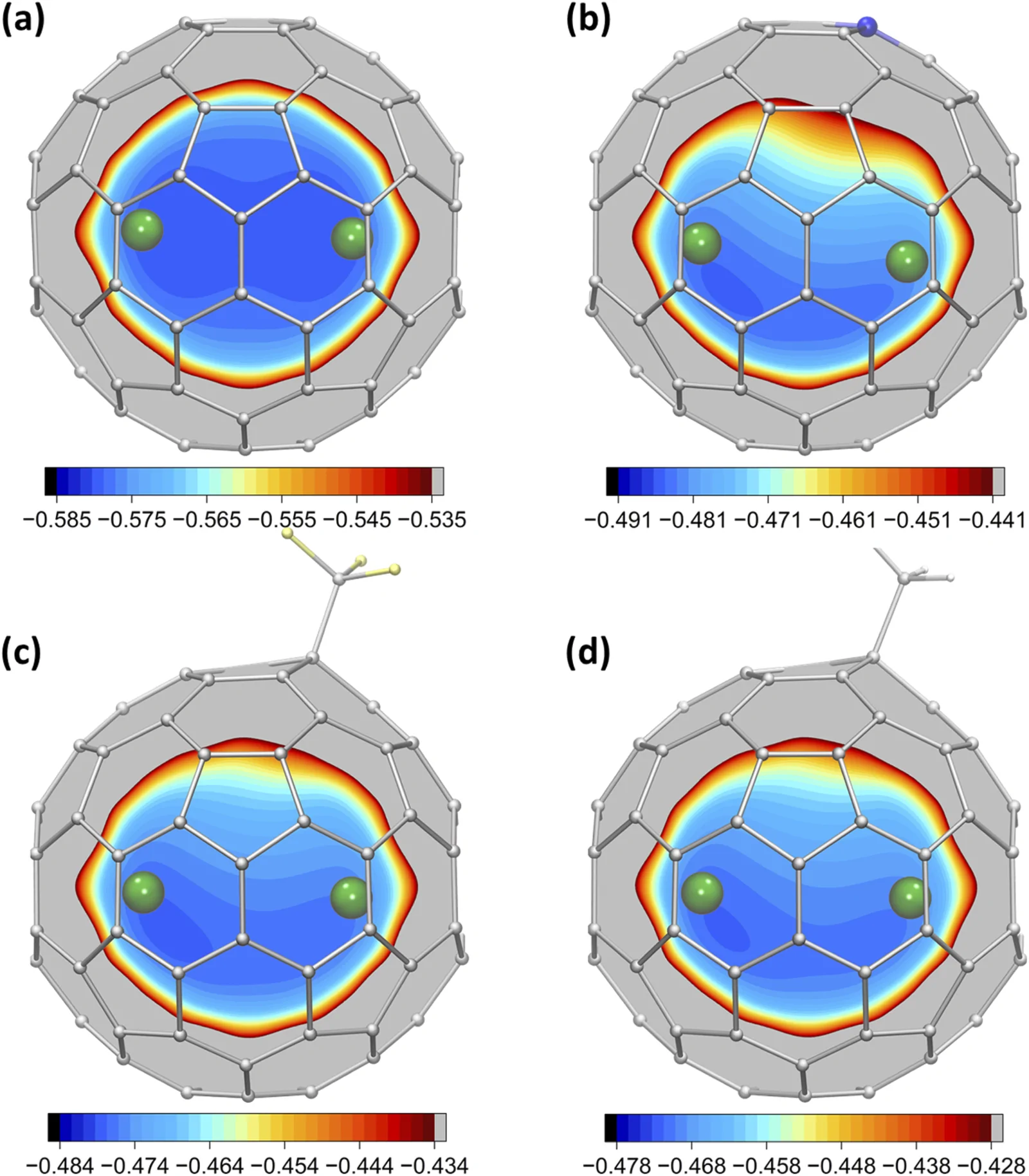
Electrostatic potential (ESP) distribution inside fullerene cages of (a) C_80_^6−^, (b) C_79_N^5−^, (c) C_80_(CF_3_)^5−^, and (d) C_80_(CH_2_Ph)^5−^. ESP values are in atomic units; ESP maps are calculated for the plane, comprising N or C-sp^3^ atoms and two metal atoms in respective di-EMFs; positions of metal atoms in di-EMFs are shown to guide the eye.

The ligand field experienced by lanthanide atoms in di-EMF can be divided into two contributions. The M–M bonding orbital localizes the electron density between metal atoms, which produces a highly axial contribution to the ligand field.^10,28^ The latter should be somewhat overestimated in our modelling as we populate the orbital with two electrons for CASSCF calculations, but this overestimation is balanced by the known tendency of CASSCF to underestimate the LF splitting in EMFs because of the lack of dynamic correlation.^93–95^ On the other hand, the metal interaction with the fragment of the fullerene cage, acting essentially as an extended and curved π-ligand, gives largely a non-axial contribution to the LF.^28,29,31^ The *η*^6^ coordination type of lanthanides, realized in di-EMFs with the *I*_h_-C_80_ cage (Fig. 1), usually does not produce a strongly axial ligand field in heavy-lanthanide complexes. Computational analysis of organometallic complexes with cyclic ligands demonstrated that the benzene ring is not compatible with strong magnetic axiality,^96,97^ and Dy complexes with the benzene ligand showed poor SMM properties.^98–101^ Besides, nitrogen in M_2_@C_79_N or C-sp^3^ atoms in M_2_@C_80_(CF_3_) and M_2_@C_80_(CH_2_Ph), albeit remote from metal positions, also add a non-axial contribution to the ligand field. The latter can be illustrated with the help of electrostatic potential distribution inside the carbon cage in respective metal-free anions (Fig. 9). Whereas the ESP around metal positions is quite symmetric inside the non-functionalized *I*_h_-C_80_^6−^, exohedral addition of CF_3_ or CH_2_Ph groups introduces visible distortion of the ESP distribution, which is most pronounced when one cage carbon is substituted by nitrogen in azafullerene C_79_N^5−^. The predominance of the axial contribution stemming from the metal–metal bond over the non-axial influence of the cage can be seen from the Ising ground states found for both metals: Kramers doublet (KD) with *J*_z_ = ±15/2 and a pseudospin *g*_z_ of 19.8–19.9 for Dy and quasi-doublet with *J*_z_ = ±6 and a pseudospin *g*_z_ of 17.9–18.0 for Tb. Dy and Tb also have comparable overall LF splitting near 890–980 cm^−1^ as well as gaps between the ground states and the first excited doublets of 180–290 cm^−1^. However, the variation of these values with molecular structure is much more pronounced in the Dy series than in the Tb analogs.

A strong difference between Dy and Tb becomes apparent when one compares compositions of higher LF states and analyzes weights of ligand field parameters with different ranks in the total LF splitting (Fig. 8). While the LF states of Tb have an almost pure *m*_J_ composition with significant mixing only at the highest energies, for Dy the mixing starts already in the first excited KD (KD2) and becomes especially strong for KD3 and KD4, while tending to restore the purity of the *m*_J_ representation towards higher energies (Fig. 8). The LF of Tb is dominated by axial parameters (mainly *B*_0_^2^, but also *B*_0_^4^ and *B*_0_^6^), with their total weight in the LF splitting amounting to 75% for [Tb_2_@C_80_]^−^ and 64–67% for other Tb di-EMFs. In the Dy series, the weight of axial terms is smaller by 15–25% and varies from 53% in [Dy_2_@C_80_]^−^, through 50% for Dy_2_@C_80_(CH_2_Ph) and 42% in Dy_2_@C_80_(CF_3_) to 39% in Dy_2_@C_79_N. Importantly, this reduction of axiality in the Dy series correlates with the susceptibility to the local-mode mechanism and thus with the low-temperature SMM behavior.

The reduced weight of axial terms in Dy is replaced by hexacontadecapolar LF parameters (*B*_q_^6^), whose total weight amounts to 35–37% in Dy di-EMFs *versus* only 12–15% in Tb di-EMFs. Hexadecapolar terms (*B*_q_^4^) are similarly small for both metals and contribute around 10% to the LF splitting. As Dy and Tb di-EMFs have almost identical metal-cage coordination geometries, such a strong variation of the weight of *B*_q_^6^ terms should originate from the intrinsic properties of these lanthanides. Indeed, they can be traced back to the size of the Stevens factors *θ*_*k*_ (also sometimes referred to as *α* for *θ*_2_, *β* for *θ*_4_, and *γ* for *θ*_6_), which enter the crystal field parameters as *B*_q_^*k*^ = *A*_q_^*k*^*r*^*k*^*θ*_*k*_, where *A*_q_^*k*^ is metal-independent and *r*^*k*^ is the radial integral. The relative contributions of different rank parameters can be qualitatively estimated as the ratio *θ*_*k*_*J*^*k*^/*θ*_2_*J*^2^. While for Dy the *θ*_4_*J*^4^/*θ*_2_*J*^2^ and *θ*_6_*J*^6^/*θ*_2_*J*^2^ values are 0.52 and −0.52, respectively, for Tb these values are −0.44 and 0.14 (Table S11). It indicates that in a similar chemical environment one can expect a considerably smaller weight of *B*_q_^6^ parameters for Tb than for Dy, which agrees with the results of our calculations. As hexacontadecapolar LF parameters are mainly non-axial and can be predominantly ascribed to metal-cage interactions, their larger weight for Dy suggests that its ligand field is less axial and more sensitive to the variation of the fullerene structure than that of Tb. It also suggests that the spin-phonon coupling for lateral metal modes, which are responsible for the local-mode relaxation mechanism, should be larger for Dy, and hence that its low-temperature magnetization dynamics should be more susceptible to the variation of the fullerene molecular structure.

The single-ion LF parameters discussed in this section can then be substituted into eqn (1) and used for calculations of the spin states and magnetic properties of the whole di-EMF system, as was done with considerable success in earlier works.^5,9,10,16,18,28^ More refined treatment should take into consideration more complex forms of exchange interactions and would require *ab initio* calculations beyond a single-ion model, which is at the very limits of the current computational capabilities and requires a separate dedicated study, going beyond this work.

## Conclusions

We performed a detailed study of how the fullerene cage and its derivatization affect the SMM behavior of endohedral lanthanide dimers with single-electron M–M bonds. While earlier studies of such dimers utilized either azafullerenes M_2_@C_79_N or radical adducts M_2_@C_80_(CH_2_Ph) and M_2_@C_80_(CF_3_), here we succeeded in the isolation of [Dy_2_@*I*_h_-C_80_]^−^ and [Tb_2_@*I*_h_-C_80_]^−^ anions with an unperturbed carbon cage. The synthesis is based on the oxidation of an anionic metallofullerene mixture by molecular iodine, as I_2_ does not react with [M_2_@*I*_h_-C_80_]^−^, but oxidizes anions of other metallofullerenes. [Dy_2_@*I*_h_-C_80_]^−^ is thus a rare example of a metallofullerene, which can be obtained without the use of chromatography. The study of the SMM behavior of [Dy_2_@*I*_h_-C_80_]^−^, [Tb_2_@*I*_h_-C_80_]^−^, and Dy_2_@C_80_(CF_3_), which was also synthesized and structurally characterized for the first time in this work, was performed by using DC and AC magnetometry. The role of intermolecular interactions in the quantum tunneling of magnetization was evaluated by field dependence and magnetic dilution using Dy_2_@C_80_(CF_3_) for a case study. These new data were combined with the results of earlier studies to compare the isostructural series of Dy_2_ and Tb_2_ dimetallofullerenes, comprising M_2_@C_79_N, M_2_@C_80_(CH_2_Ph), M_2_@C_80_(CF_3_), and [M_2_@*I*_h_-C_80_]^−^.

We found that the derivatization of the host fullerene cage does matter for lanthanide dimers with metal–metal bonds, and the higher symmetry of the fullerene host in [M_2_@*I*_h_-C_80_]^−^ appears to be instrumental in increasing the blocking temperature of magnetization. The effect is more pronounced for Dy_2_ dimetallofullerenes, indicating that relaxation characteristics of the Dy_2_ dimer are more sensitive to the environment than in Tb_2_ analogs. Both Dy_2_ and Tb_2_ series demonstrate a significant variation of QTM times, but the order of compounds in the two series is different, and there is no simple correlation between the derivative type and the QTM time. Another important difference between Dy_2_ and Tb_2_ dimetallofullerenes is that the former show an intermediate relaxation regime between the temperature ranges dominated by the QTM and Orbach mechanisms, while such an intermediate region is absent in the Tb_2_ series. We ascribe this intermediate regime to the local-mode mechanism, presumably induced by lateral metal-based vibrations. This mechanism causes a large variation of blocking temperatures in the Dy_2_ series and unconventional shapes of zero-field-cooled magnetization curves for Dy_2_@C_80_(CF_3_) and Dy_2_@C_79_N. The width of hysteresis is also strongly affected by the derivatization of the fullerene cage.

We showed that the higher sensitivity of magnetization relaxation in the Dy_2_ series to the molecular structure of the fullerene host and lateral metal modes correlates with the stronger variation and lower axiality of the single-ion magnetic anisotropy in Dy di-EMFs than in Tb analogs. In particular, the ligand field experienced by Dy ions has a much higher weight of non-axial hexacontadecapolar LF parameters, while the LF of Tb is predominantly axial. This difference can be traced back to an intrinsic property of the lanthanide ions, namely the size of their Stevens factors, which suggests that in isostructural compounds the contribution of hexacontadecapolar terms for Tb should be several times smaller than for Dy.

Altogether, these findings demonstrate that the chemical modification of the carbon cage can become a useful tool to tune the magnetic properties of encapsulated metal dimers with metal–metal bonds. As addition of exohedral chemical groups turns C-sp^2^ atoms into C-sp^3^, it modifies the π-system topology and can change the local coordination geometry of metal atoms and the potential energy surface for their lateral motion inside the fullerene cage. This will potentially lead to changes in the ligand-field splitting and vibrational properties and thus affect the spin-phonon coupling. Our results show that Dy di-EMFs should be more susceptible to this kind of tuning than Tb di-EMFs.

## Author contributions

M. F. S. B. performed magnetic measurements; W. Y. synthesized Dy di-EMFs; Y. W. synthesized Tb di-EMFs; S. M. A. performed *ab initio* calculations, F. L. supervised synthesis of EMFs and performed single-crystal X-ray diffraction analysis; A. A. P. conceived the study, performed DFT calculations, and drafted the manuscript with contributions from other co-authors. All co-authors participated in the discussion and editing of the manuscript.

## Conflicts of interest

There are no conflicts to declare.

## Supplementary Material

SC-OLF-D6SC04567G-s001

SC-OLF-D6SC04567G-s002

## Data Availability

CCDC 2334090 contains the supplementary crystallographic data for this paper.^102^ The data supporting this article have been included as part of the supplementary information (SI). Supplementary information: experimental and computational details, HPLC separation, MS spectra, additional computational results, crystallographic details, and results of relaxation measurements (PDF). See DOI: https://doi.org/10.1039/d6sc04567g.
