# Supplementary material for: Relaxation of magnetization in Dy2 and Tb2 dimers with single-electron lanthanide–lanthanide bonds: the effect of cage perturbation and its ultimate symmetry
Source: Chem Sci. 2026 Aug 19;17(40):19506–22. doi: 10.1039/d6sc04567g (PMC13532669; doi:10.1039/d6sc04567g)
Supplement: Supplementary file 1 [file SC-017-D6SC04567G-s001.pdf]

Supplementary Information (SI) for Chemical Science.
This journal is © The Royal Society of Chemistry 2026

Relaxation of magnetization in Dy_2 and Tb_2 dimers with single-electron lanthanide-lanthanide bond: The effect of the cage perturbation and its ultimate symmetry

Matheus Felipe de Souza Barbosa,[$1] Wei Yang,[$1] Yaofeng Wang,[$1] Georgios Velkos,[1] Stanislav M. Avdoshenko,[*1] Fupin Liu,[*2] Alexey A. Popov[*1]

Supporting Information

Experimental details S2

Synthesis and separation of $Dy_2@C_{79}N$ S3

Synthesis and separation $Dy_2@C_{80}(CF_3)$ S5

Oxidation of EMF anions with iodine S7

SC-XRD analysis of $Dy_2@C_{80}(CF_3)$ S10

Relaxation of magnetization S13

BS-DFT calculations S21

LF splitting and Steven factors S22

References S23

Experimental and computational details

HPLC separation was performed with toluene as an eluent, employing semipreparative COSMOSIL Buckyprep chromatographic columns (Nacalai Tesque) and Agilent 1260 Infinity II LC System. Recycling HPLC separation was performed using the Sunflow 100 system (SunChrome).

Mass spectrometry: Laser desorption/ionization time-of-flight (LDI-TOF) mass-spectra were measured with a Bruker autoflex mass-spectrometer.

UV-Vis spectrometry: UV-vis-NIR absorption spectra were measured in toluene solution at room temperature with Shimadzu 3100 spectrophotometer.

X-ray diffraction. Single crystals were obtained by co-crystallization of $Dy_2@C_{80}(CF_3)$ in toluene with nickel octaethylporphyrin (NiOEP) in benzene. Single crystal X-ray diffraction data collection was carried out at 100 K at the BESSY storage ring (BL14.2, Berlin-Adlershof, Germany).[1] XDSAPP2.0 suite was employed for data processing.[2, 3] The structure was solved by direct methods and refined by SHELXL-2018.[4] Hydrogen atoms were added geometrically and refined with a riding model.

Magnetic measurements of powder samples were performed using a Quantum Design VSM MPMS3 magnetometer. For DC measurements, the samples were drop-casted onto the quartz sample-holder from CS_2 or acetone solution. Magnetization decay curves were measured after the samples were first magnetized at 7 Tesla and then the field was quickly ramped to 0 T or another required field. The decay curves were fitted with stretched exponential function:

$$M(t) = M_{eq} + (M_0 - M_{eq}) \exp\left[-\left(\frac{t}{\tau_M}\right)^{\beta}\right]$$

Where M_{eq} and M_0 are the equilibrium and initial magnetizations, respectively, τ_M is a characteristic relaxation time and β is a parameter, characterizing distribution of relaxation rates in the sample. For a single-exponential decay, β = 1. When relaxation time is very long, M_{eq} and τ_M become correlated, and their simultaneous determination from the fit is not reliable (unless the measurable time is extremely long). In such cases, we measured M_{eq} independently and used it as a fixed parameter.

In AC magnetometry studies, the in-phase and out-of-phase susceptibility, $\chi'(\nu)$ and $\chi''(\nu)$, were measured as a function of oscillation frequency ν and then fitted with the generalized Debye model to determine relaxation times τ_{M} and parameters α:

$$\chi'(\nu) = \chi_S + (\chi_T - \chi_S)\frac{1 + (\nu\tau_{\mathrm{M}})^{1-\alpha}\sin\left(\frac{\pi\alpha}{2}\right)}{1 + 2(\nu\tau_{\mathrm{M}})^{1-\alpha}\sin\left(\frac{\pi\alpha}{2}\right) + (\nu\tau_{\mathrm{M}})^{2-2\alpha}}$$

$$\chi''(\nu) = (\chi_T - \chi_S)\frac{(\nu\tau_{\mathrm{M}})^{1-\alpha}\cos\left(\frac{\pi\alpha}{2}\right)}{1 + 2(\nu\tau_{\mathrm{M}})^{1-\alpha}\sin\left(\frac{\pi\alpha}{2}\right) + (\nu\tau_{\mathrm{M}})^{2-2\alpha}}$$

DFT calculations were performed using the *Orca* package.[5, 6] All-electron DFT calculations can give ambiguous results for lanthanides because of the non-single-determinant wavefunction. To avoid these problems, we used 4f-in-core effective core potentials of ECP*XX*MWB type by Dolg et al. with corresponding ECP*XX*MWB-II basis sets.[7, 8] For C, H, and F atoms, def2-TZVPP basis was used.[9] For broken-symmetry DFT calculations of Gd di-EMFs, PBE0 functional with DKH scalar-relativistic correction were used with full-electron SARC-DKH-TZVP basis set for Gd and SARC-adapted def2-TZVP basis for other elements.[10]

CASSCF/RASSI-SO calculations of LF energies and wave functions of Dy^{3+} and Tb^{3+} LF multiplets in di-EMFs molecules were performed using the quantum chemistry package OpenMOLCAS[11] and SINGLE_ANISO module.[12] The basis sets were ANO-RCC-VTZP for Dy and Tb and ANO-RCC-VDZP for other elements. Calculations were performed for di-EMF anions, one lanthanide was replaced with Y.

Synthesis and isolation of $Dy_2@C_{79}N$

Dy-EMFs were synthesized by an arc-discharge process. Carbon rods, each filled with 1.67 g of Dy_2O_3 powder and 0.83 g of graphite powder (molar ratio of Dy/C = 1:7.5), were vaporized under a He/N_2 atmosphere (180 mbar/10 mbar). The soot was collected and refluxed in carbon disulfide (CS_2) under an argon atmosphere for 12 h. Totally, 100 rods were vaporized and ca. 5 g of crude fullerene extract was obtained (ca. 50 mg per rod). CS_2 crude extract was dried and re-dissolve in toluene. The crude toluene solution was reacted with dried diamino silica gel (DASG). Empty fullerenes, and many Dy-EMFs reacted with amino groups and were trapped by DASG, whereas less reactive $Dy_3N@C_{2n}$ clusterfullerenes mainly remained in solution (filtrate, Figure S1b). The DASG with immobilized fullerenes was then filtered and washed with CS_2, which resulted in the release of Dy-EMFs, whereas empty fullerenes mainly remained trapped (Figure S1c). In ref. [13] we isolated two isomers of $Dy_2C_2@C_{88}$ from the mixture of released fullerenes. In this work, $Dy_2@C_{79}N$ was isolated from the same mixture as shown in Figure S2.

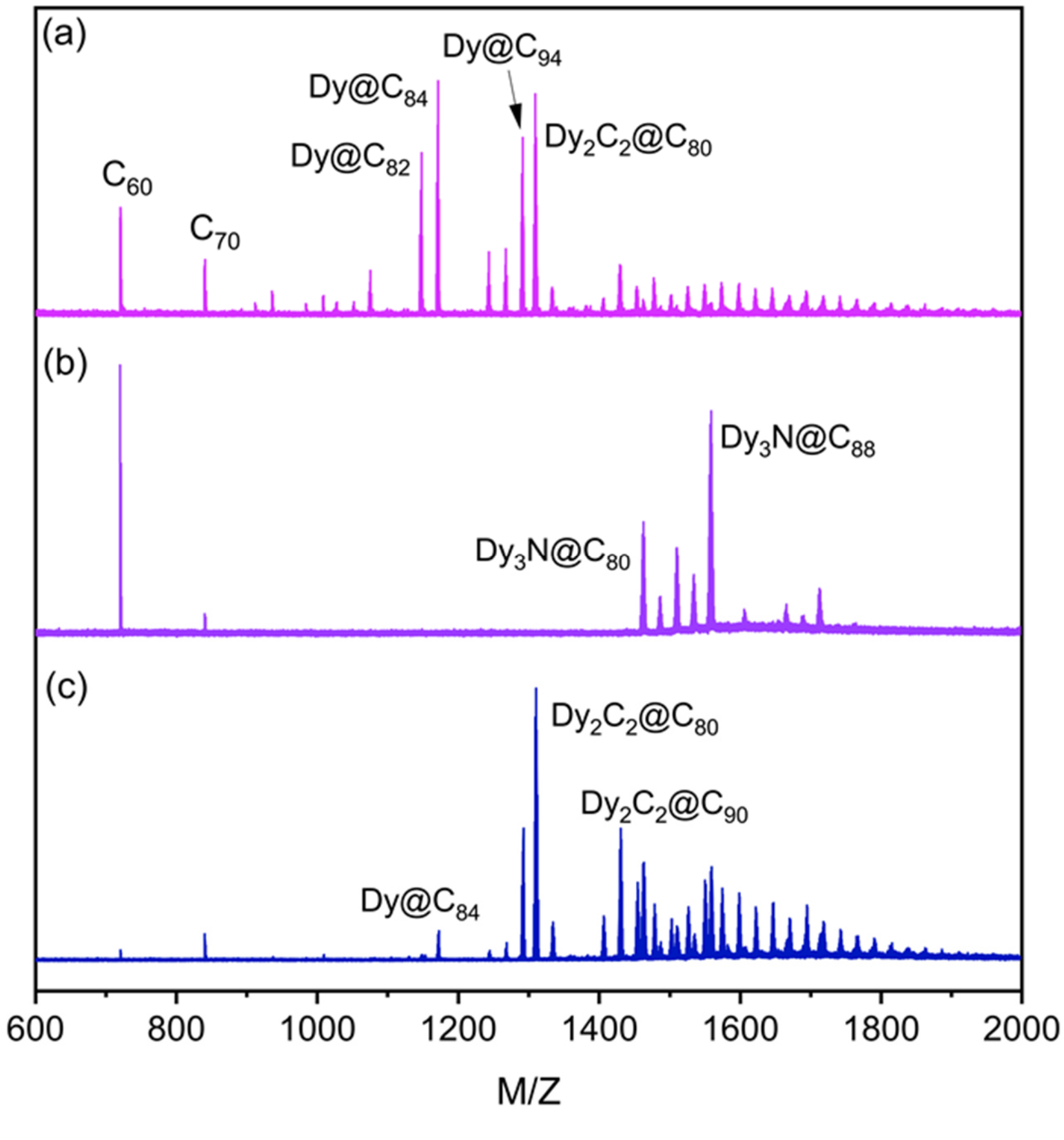

Figure S1. Positive-ion LDI mass spectra (a) crude toluene solution before reaction with DASG; (b) filtered toluene solution after reaction with DASG, main EMFs are nitride clusterfullerenes $Dy_3N@C_{2n}$; (c) CS_2 wash of spent DASG, which released trapped EMFs. Note that intensity in mass-spectra does not necessarily correlates with concentration, but also strongly depends on the efficiency of ionization. MS does not allow one to distinguish $Dy_2@C_{2n}$ from $Dy_2C_2@C_{2n-2}$, so their assignment is arbitrary. For instance, $Dy_2C_2@C_{80}$ can also be $Dy_2@C_{82}$, or both compounds can be present.

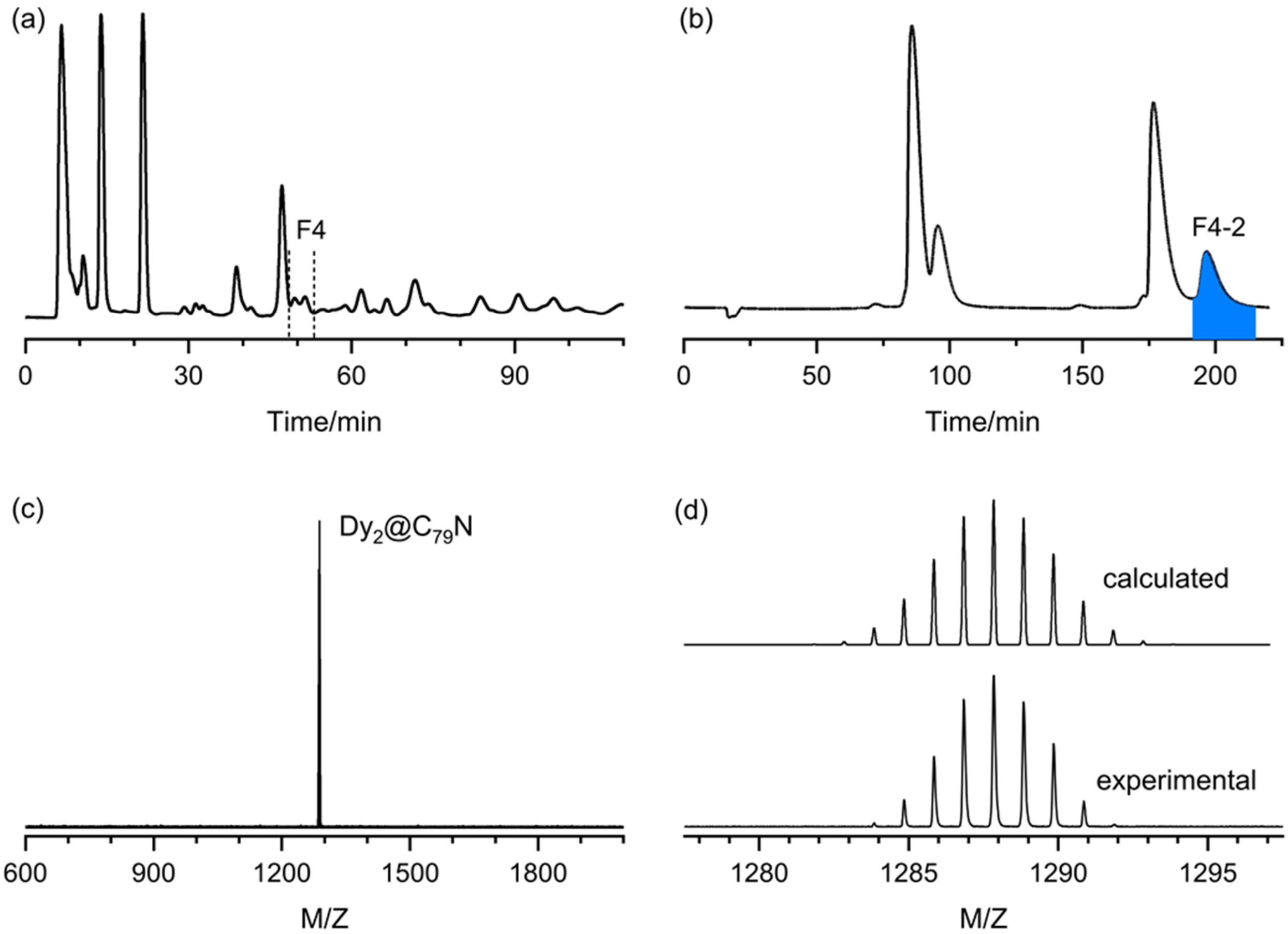

Figure S2. HPLC separation of $Dy_2@C_{79}N$. (a) The first stage HPLC chromatogram of extract recovered from DASG on a 2×Buckyprep column (Φ = 10 mm × 250 mm, flow rate 5 mL/min), $Dy_2@C_{79}N$ is found in fraction **F4**; (b) the second stage recycling HPLC chromatogram of fraction **F4** on a Buckyprep-M column (Φ = 10 mm × 250 mm, flow rate 1 mL/min). Fraction **F4-2** is $Dy_2@C_{79}N$.The HPLC conditions were: eluent = toluene; detecting wavelength = 310 nm. Reflection negative-ion LDI mass spectrum of $Dy_2@C_{79}N$ (c) with isotopic distribution of main peaks (d).

Synthesis and isolation of $Dy_2@C_{80}(CF_3)$

Fullerene-containing soot left after CS_2 extraction was extracted with hot DMF for 24 hours, which resulted in solution of Dy-EMF anions. Part of this extract was used for the synthesis of $Dy_2@C_{80}(CF_3)$ described here, while another part was used for the isolation of $[Dy_2@I_h\text{-}C_{80}]^-$.

Scheme of the reaction between EMF anions and Umemoto reagent II with formation of $EMF(CF_3)_n$ adducts:

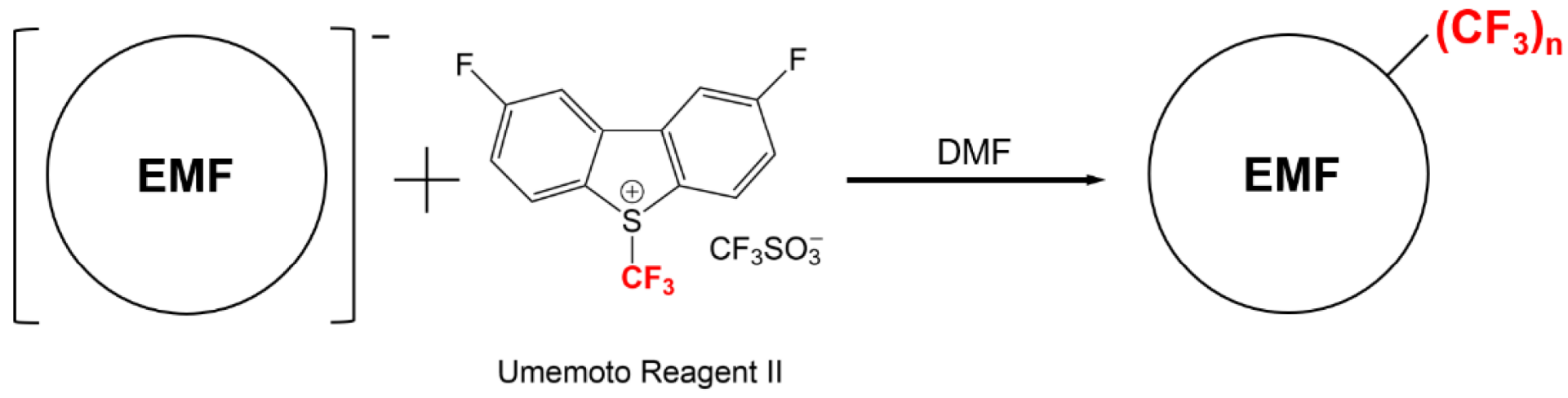

Reaction proceeds at room temperature. Under optimized ratio of reagents (see Figure S3 for the optimization), the reagent shows enhanced selectivity towards $[Dy_2@I_h\text{-}C_{80}]^-$ with formation of $Dy_2@I_h\text{-}C_{80}(CF_3)$ as the main product. Separation of $Dy_2@I_h\text{-}C_{80}(CF_3)$ from other $EMF(CF_3)$ adducts is shown in Figure S4.

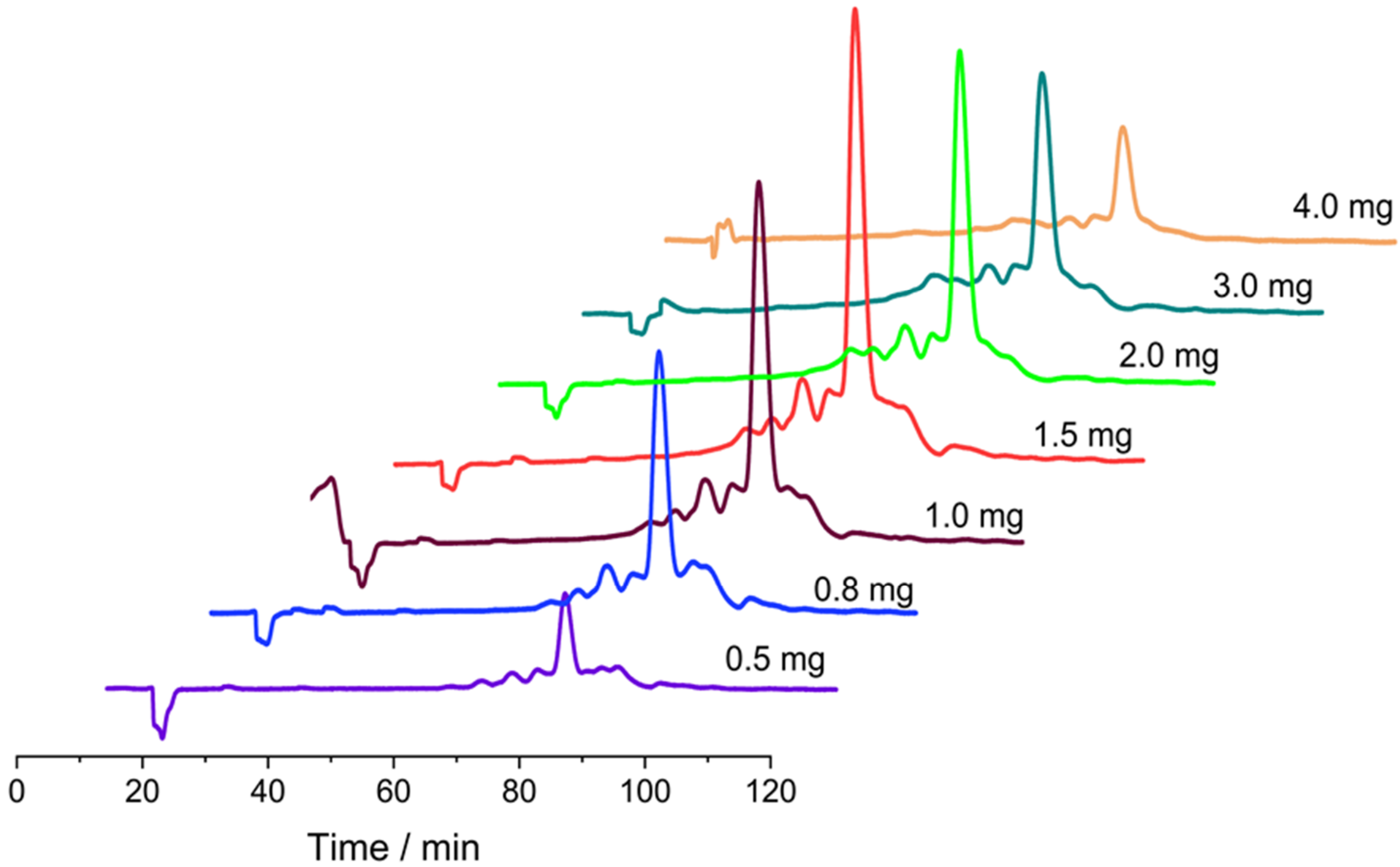

Figure S3. HPLC profiles of toluene-soluble products formed in the reaction of 5 mL aliquots of Dy-EMF extract in DMF with different amounts of Umemoto reagent II. $Dy_2@I_h\text{-}C_{80}(CF_3)$ is the main component of the most prominent fraction. 10×250 mm Buckyprep column; flow rate 2.0 mL/min; toluene as eluent.

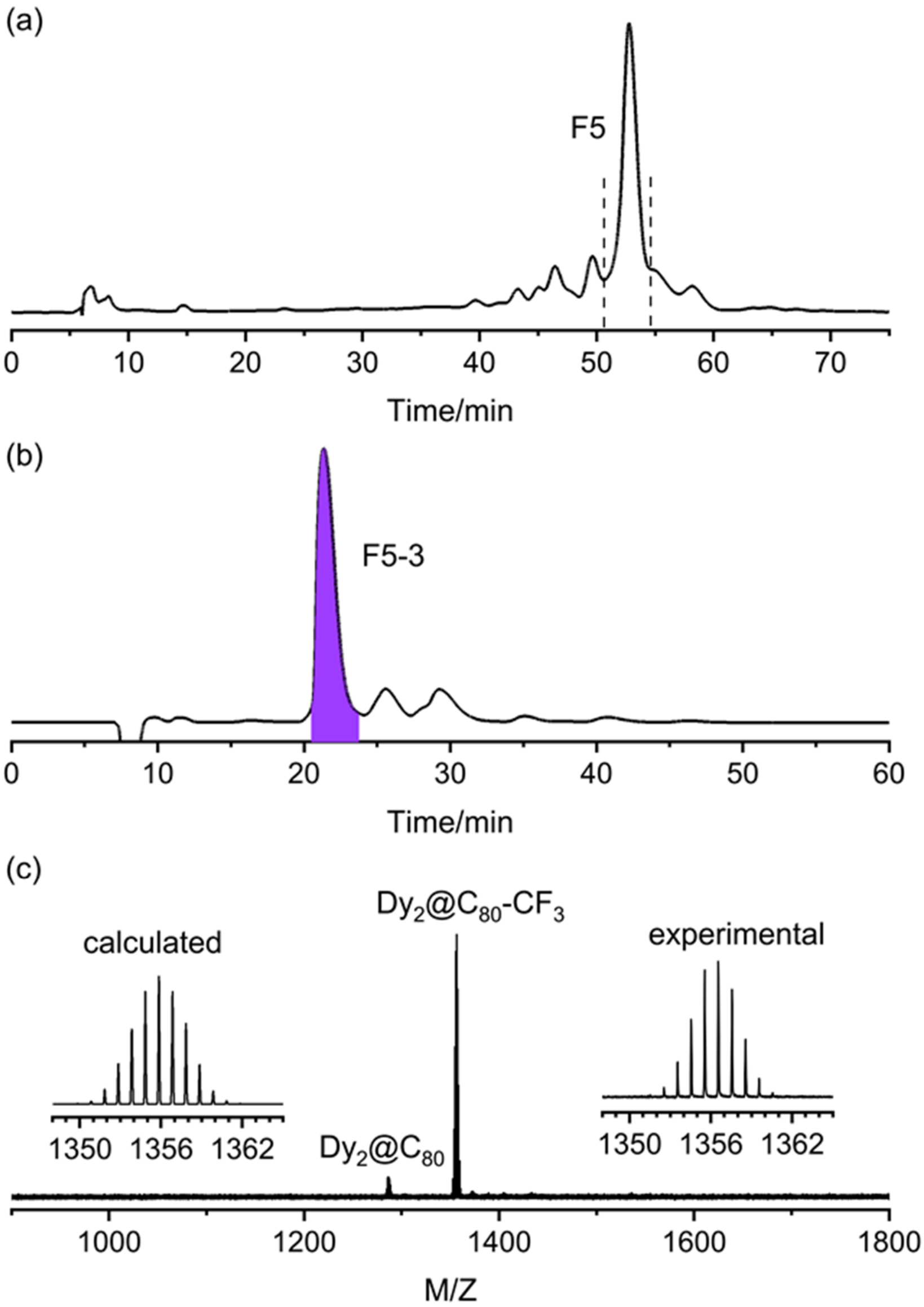

Figure S4. HPLC separation of $Dy_2@I_h$-$C_{80}(CF_3)$. (a) The 1st-step linear HPLC isolation (2×Buckyprep columns, flow rate 5 ml/min), fraction 5 contains the target compound. (b) the 2nd-step linear HPLC isolation (2×Buckyprep-D columns, flow rate 4 ml/min), fraction F5-3 is $Dy_2@I_h$-$C_{80}(CF_3)$. (c) reflection positive-ion MALDI (TPBD as matrix) mass spectrum of F5-3, insets show experimental and calculated isotopic patterns.

Table S1. DFT-computed electron affinity of Tb-EMFs and Dy-EMFs in gas phase (eV) and relative reduction potential in acetone (in V)

	EA (gas)		Δ*EA*(gas)		$\Delta E^{0/-}$(acetone)	
EMF	M = Tb	M = Dy	M = Tb	M = Dy	M = Tb	M = Dy
$M@C_s(6)-C_{82}$	3.323	3.325	−0.473	−0.472	−0.472	−0.471
$M@C_{2v}(9)-C_{82}$	3.440	3.442	−0.357	−0.355	−0.362	−0.360
$M_2@D_{5h}(6)-C_{80}$	3.419	3.418	−0.378	−0.379	−0.365	−0.367
$M@C_{2v}(5)-C_{80}$	3.486	3.487	−0.311	−0.310	−0.304	−0.305
$M_2@D_{3h}-C_{78}$	3.487	3.487	−0.310	−0.310	−0.295	−0.295
$M@C_{3v}(8)-C_{82}$	3.771	3.773	−0.026	−0.024	−0.033	−0.032
$M_2@I_h(7)-C_{80}$	3.797	3.797	0.000	0.000	0.000	0.000

Computations performed at the PBE/def2-TZVPP level with Dolg's 4f-in-core effective potential for Dy and Tb and C-PCM solvation correction for acetone, the values are in eV for *EA* and in V for reduction potentials $\Delta E^{0/-}$.

Analysis of the precipitate formed after oxidation of Dy-EMF anions by iodine

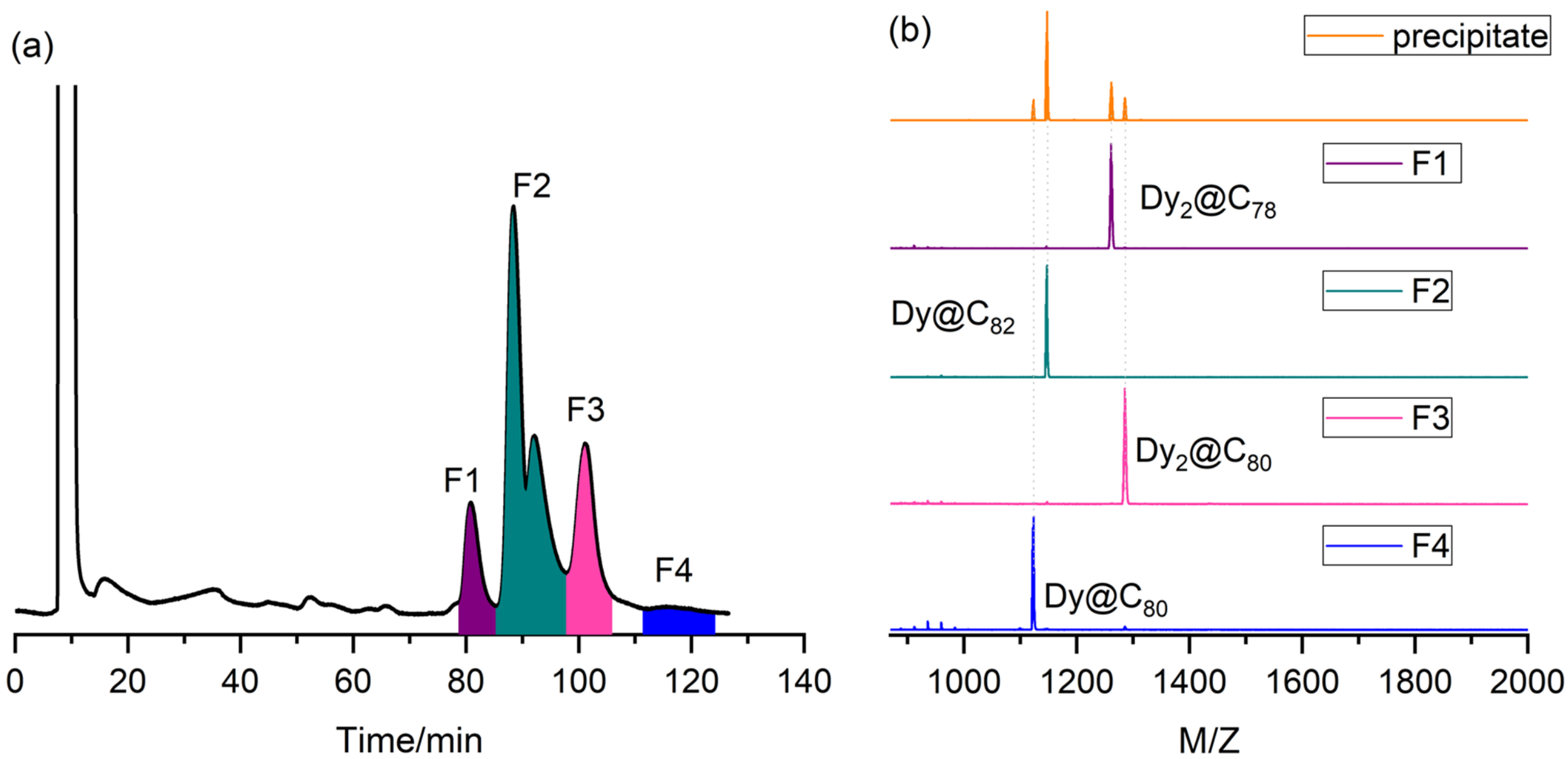

Figure S5. (a) HPLC trace of the precipitate after oxidation of Dy-EMF anions by iodine. (b) MALDI mass-spectra of individual fractions.

Oxidation of Tb-EMF anions by iodine

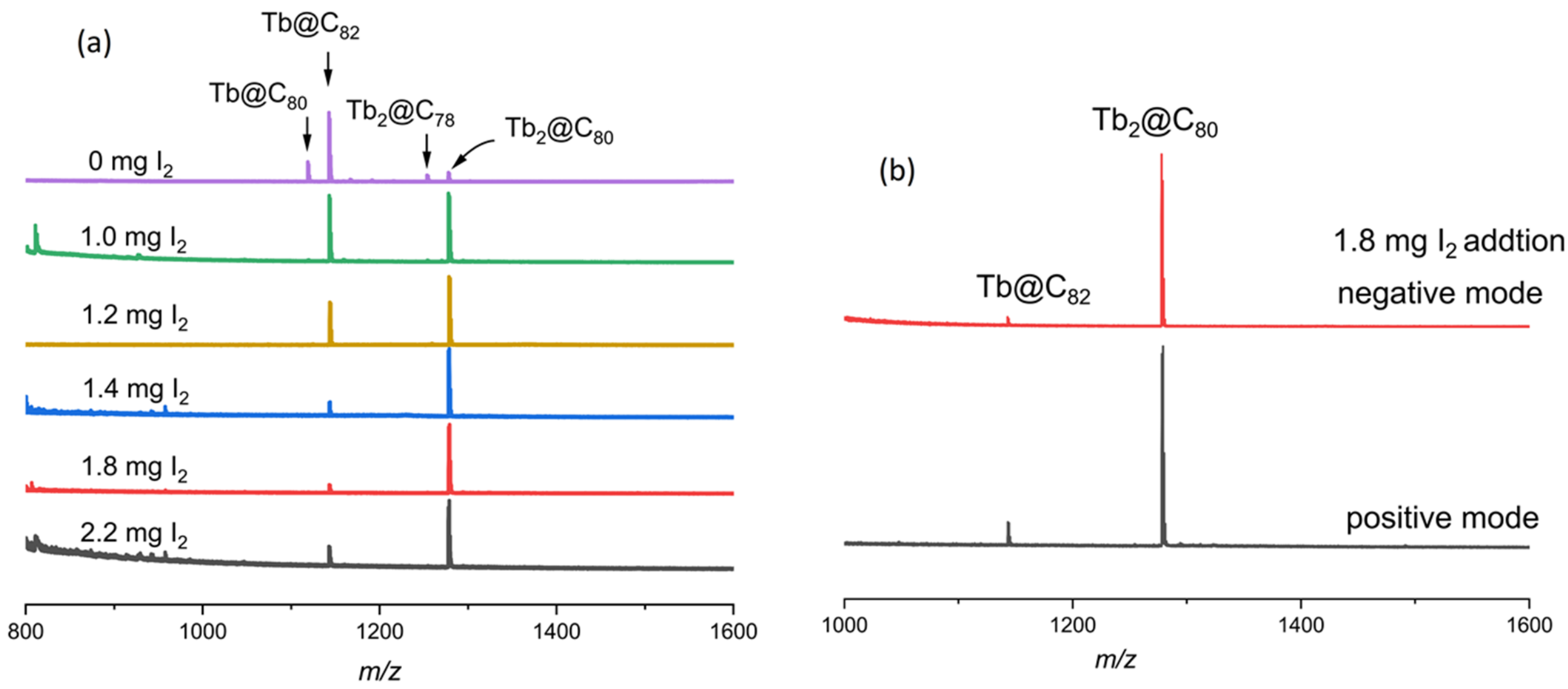

Figure S6. (a) Mass-spectra of acetone/CS_2 solution after addition of different amount of I_2 to 10 mL aliquots of anionic Tb-EMF extract in acetone/CS_2. The signal of Tb@C_{82} decreases relative to Tb_2@C_{80} but does not disappear completely; by addition of a large amount of iodine, target [Tb_2@I_h-C_{80}]$^-$ also precipitates. (b) Positive and negative ion mass-spectra of the acetone solution left after addition of 1.8 mg of I_2.

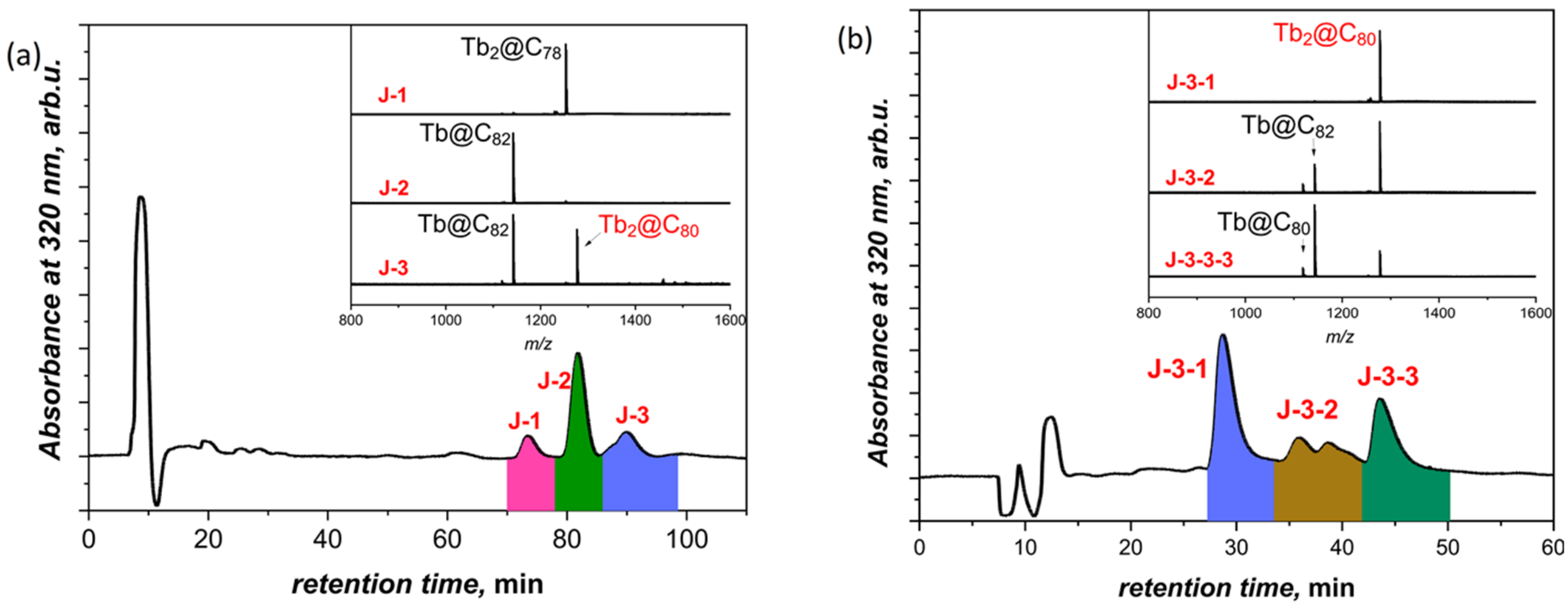

Figure S7. (a) HPLC profile for separation of precipitate after oxidation of Tb-EMF anions by iodine (Buckyprep column, flow rate: 2 mL/min); Insert shows positive ionization MALDI-TOF mass spectra of fraction J-1, J-2 and J-3. (b) HPLC profile for further separation of fraction J-3 (Buckyprep-D column, flow rate: 2 mL/min). Insert shows positive ionization MALDI-TOF mass spectra of fraction J-3-1, J-3-2 and J-3-3. Tb_2@D_{3h}-C_{78} and Tb_2@D_{5h}-C_{80} are fractions J-1 and J-3-1, respectively.

Since complete removal of $[Tb@C_{82}]^-$ from $[Tb_2@I_h\text{-}C_{80}]^-$ could not be accomplished by reaction with iodine, we used chromatography. Anionic Tb-EMF extract was first reacted with sufficient amount of iodine to remove easily-oxidizable anions (1.2 mg per 10 mL extract in 4:1 acetone/CS_2). Solution with $[Tb@C_{82}]^-$ and $[Tb_2@I_h\text{-}C_{80}]^-$ anions was then separated by HPLC with acetone as eluent (Figure S8).

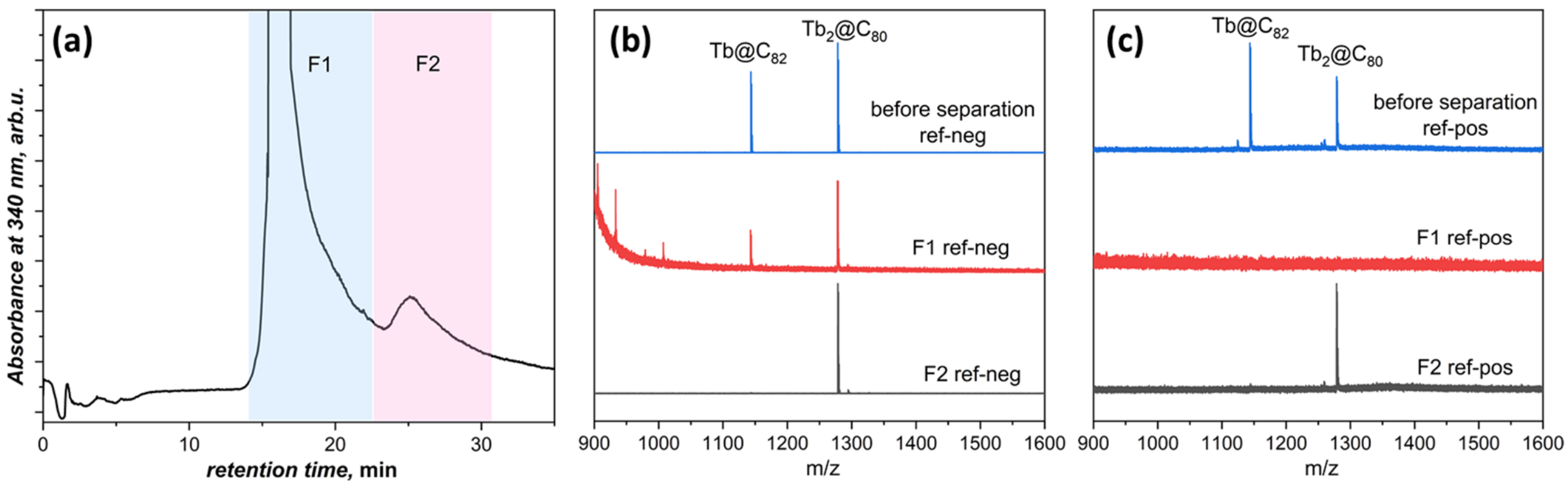

Figure S8. (a) HPLC separation of a mixture of $[Tb@C_{82}]^-$ and $[Tb_2@I_h\text{-}C_{80}]^-$, 2 x Poroshell columns, acetone as eluent. (b, c) negative and positive ion MALDI mass-spectra of the sample before separation and separated fractions. $[Tb_2@I_h\text{-}C_{80}]^-$ without admixture of $Tb@C_{82}$ is found in F2. F1 seems to contain non-fullerene components.

Table S2. Crystal data on $Dy_2@I_h(7)-C_{80}(CF_3)$

**Crystal**	**$2Dy_2@I_h(7)-C_{80}(CF_3)\cdot 4NiOEP\cdot 1.58C_7H_8\cdot 0.42C_6H_6$**
**Formula**	$C_{319.58}H_{195.57}F_6N_{16}Nd_4Ni_4$
**Formula weight**	5258.22
**Color, habit**	Black, block
**Crystal system**	monoclinic
**Space group**	*C*2/*c*
***a*, Å**	43.010(9)
***b*, Å**	41.210(8)
***c*, Å**	23.460(5)
***α*, deg**	90
***β*, deg**	96.94(3)
***γ*, deg**	90
**Volume, Å^3**	41277(15)
Z	8
***T*, K**	100
**Radiation (λ, Å)**	Synchrotron Radiation (0.7999)
**Unique data (R_{int})**	65173 (0.0987)
**Parameters**	4978
**Restraints**	5966
**Observed data ($I > 2\sigma(I)$)**	52457
**$R_1{}^a$ (observed data)**	0.0845
**$wR_2{}^b$ (all data)**	0.2555
**CCDC NO.**	2334090

[a]For data with $I > 2\sigma(I)$, $R_1 = \frac{\sum||F_o|-|F_c||}{\sum|F_o|}$. [b]For all data, $wR_2 = \sqrt{\frac{\sum[w(F_o^2-F_c^2)^2]}{\sum[w(F_o^2)^2]}}$.

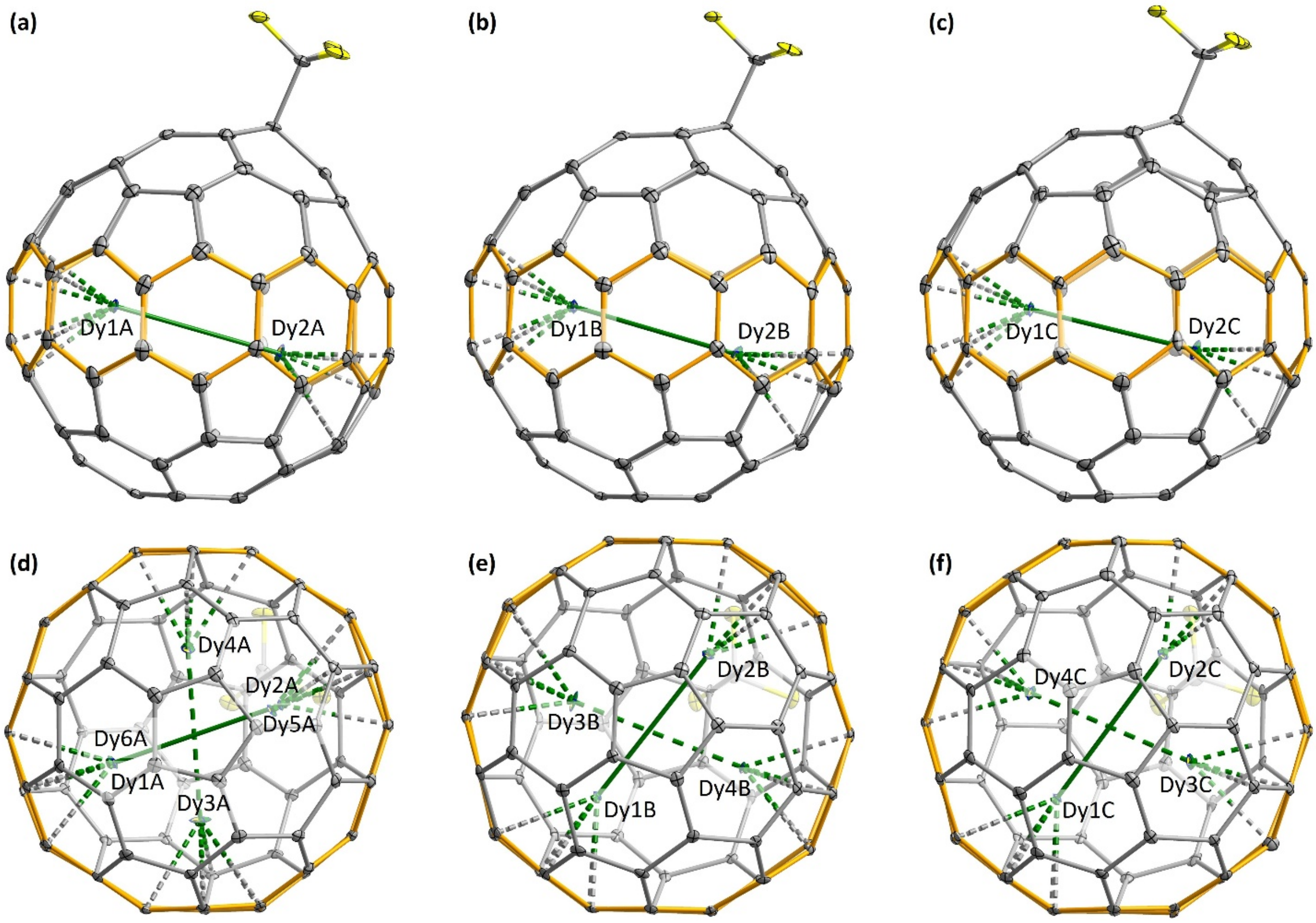

Figure S9a. Structure of $Dy_2@I_h(7)\text{-}C_{80}(CF_3)$. The displacement parameters are shown at the 10% probability level. Colour code: grey for carbon, green for Dy, and yellow for F. The belt of hexagons around which metal dimer locate is highlighted yellow. The Dy disordered sites are labeled with site occupancies of 0.55, 0.19, and 0.26 for Dy1A/Dy2A, Dy3A/Dy4A, and Dy5A/Dy6A; 0.39, and 0.11 for Dy1B/Dy2B, and Dy3B/Dy4B; 0.38 and 0.12 for Dy1C/Dy2C, and Dy3C/Dy4C, respectively.

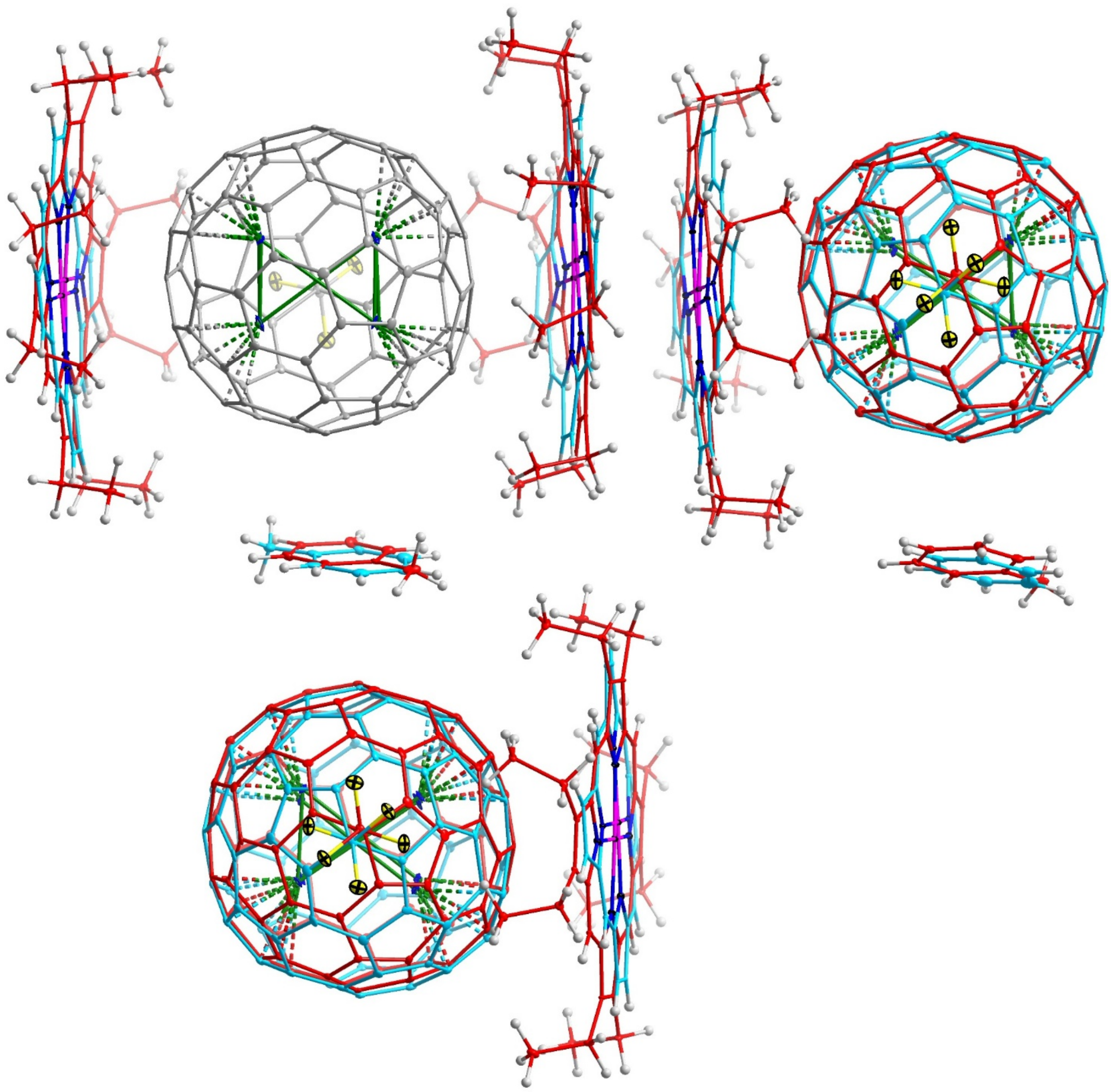

Figure S9b. The structure of $2Dy_2@I_h(7)\text{-}C_{80}(CF_3)\cdot4NiOEP\cdot1.58C_7H_8\cdot0.42C_6H_6$ with all molecules in the asymmetric unit. Color code: gray for fully ordered C, red/cyan for disordered C, white for H, blue for N, pink for Ni, yellow for F, and green for Dy. The thermal ellipsoids are set at 5% probability.

Relaxation of magnetization in $Dy_2@C_{80}(CF_3)$

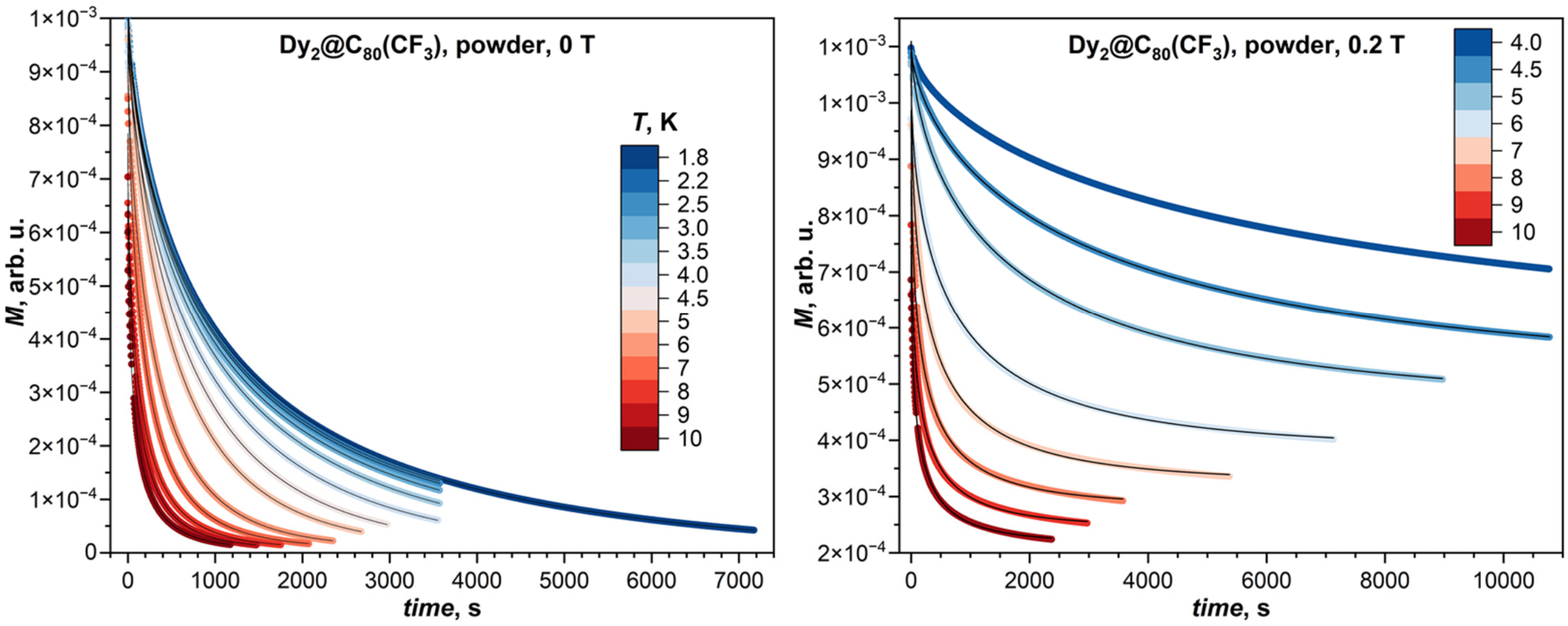

Figure S10. Magnetization decay (colored dots) and fitting with stretched exponential (black lines) of powder $Dy_2@C_{80}(CF_3)$ measured at different temperatures in zero field (left) and in the field of 0.2 T (right).

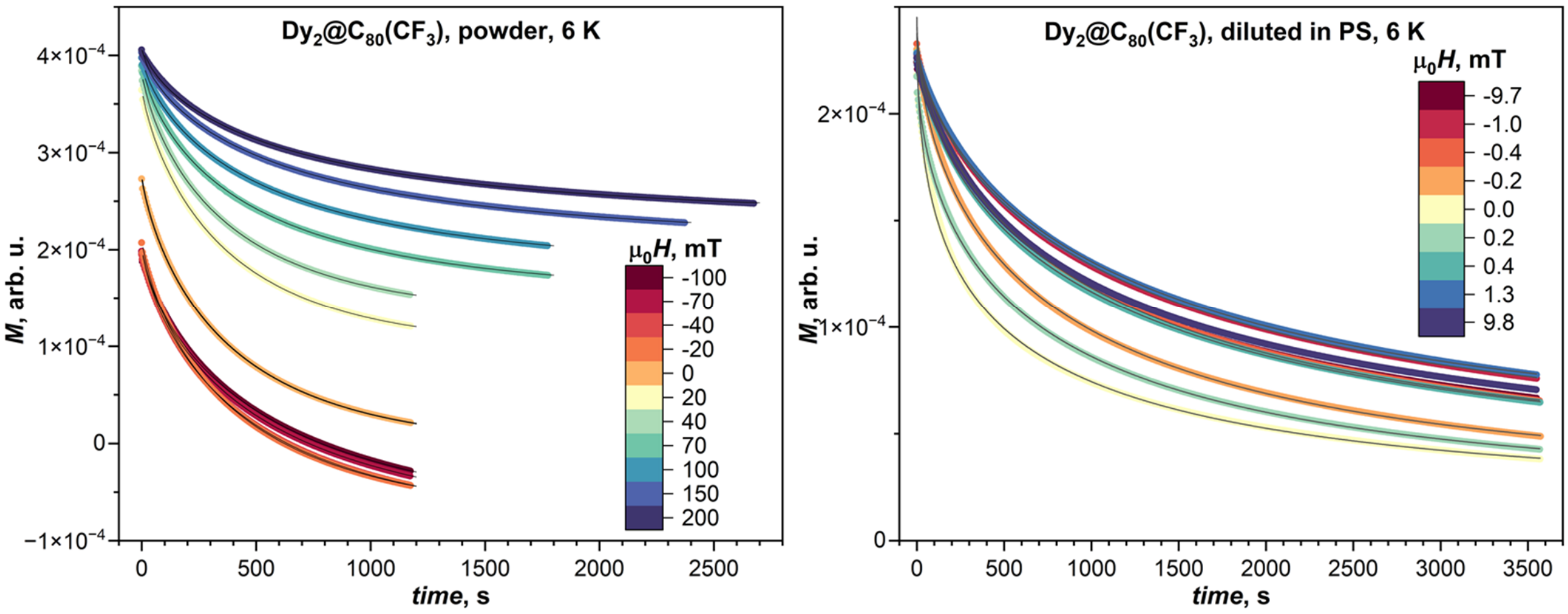

Figure S11. Magnetization decay (colored dots) and fitting with stretched exponential (black lines) of powder $Dy_2@C_{80}(CF_3)$ (left) and $Dy_2@C_{80}(CF_3)$ diluted in PS (right) measured at 6 K in different fields. Only representative curves from more than 80 decays are shown (see Tables S4-S5 for full set of fitted data).

Table S3. Magnetization relaxation times of $Dy_2@C_{80}(CF_3)$ from DC measurements at different temperatures

	0 T				0.2 T			
***T*, K**	***τ*, s**	**±**	**β**	**±**	***τ*, s**	**±**	**β**	**±**
1.8	1157.0	1.4	0.598	5E-04				
2.2	1112.5	1.2	0.634	1E-03				
2.5	1094.9	1.1	0.636	9E-04				
3.0	1067.4	1.1	0.644	1E-03				
3.5	1005.0	0.7	0.666	6E-04				
4.0	909.3	0.4	0.726	4E-04	6900.7	5.0	0.631	2E-04
4.5	715.4	0.2	0.753	2E-04	3344.2	3.4	0.633	7E-04
5	558.7	0.3	0.772	3E-04	1796.7	2.9	0.608	1E-03
6	333.4	1.4	0.727	2E-03	746.4	4.2	0.560	2E-03
7	196.7	2.1	0.646	3E-03	305.9	4.3	0.485	3E-03
8	155.2	1.8	0.617	3E-03	194.3	3.6	0.477	4E-03
9	102.8	1.5	0.564	3E-03	148.5	3.3	0.467	5E-03
10	89.3	1.3	0.558	4E-03	123.2	2.4	0.471	5E-03

Table S4. Magnetization relaxation times of $Dy_2@C_{80}(CF_3)$ at 6 K in different fields, DC measurements

**$\mu_0 H$, mT**	**τ, s**	**±**	**β**	**±**	**$\mu_0 H$, mT**	**τ, s**	**±**	**β**	**±**
**-100.0**	575.2	2.2	0.722	0.004	**26.0**	379.9	0.7	0.800	0.002
**-90.0**	568.0	2.1	0.722	0.004	**28.0**	381.2	0.8	0.798	0.002
**-80.0**	560.1	2.1	0.721	0.004	**30.0**	381.1	0.8	0.793	0.002
**-70.0**	544.7	2.5	0.723	0.005	**35.0**	385.9	0.9	0.793	0.003
**-60.0**	525.7	1.8	0.728	0.004	**40.0**	391.9	0.9	0.780	0.002
**-50.0**	503.1	1.8	0.734	0.004	**45.0**	399.6	1.1	0.778	0.003
**-45.0**	491.8	1.3	0.734	0.003	**50.0**	409.1	1.2	0.769	0.003
**-40.0**	481.7	1.3	0.743	0.003	**55.0**	420.6	1.3	0.767	0.003
**-35.0**	467.6	1.1	0.743	0.003	**60.0**	430.3	1.5	0.764	0.003
**-30.0**	455.2	1.1	0.747	0.003	**65.0**	479.5	1.5	0.709	0.003
**-25.0**	441.3	1.1	0.751	0.003	**70.0**	494.4	1.6	0.707	0.003
**-20.0**	430.3	0.8	0.756	0.002	**75.0**	509.9	1.7	0.703	0.003
**-15.0**	418.1	0.8	0.762	0.002	**80.0**	522.8	1.7	0.703	0.003
**-10.0**	408.8	0.9	0.762	0.003	**85.0**	536.8	1.8	0.704	0.003
**-5.0**	401.6	1.3	0.762	0.003	**90.0**	550.2	1.8	0.706	0.003
**-4.0**	399.8	1.7	0.761	0.004	**95.0**	561.4	1.9	0.708	0.003
**-3.0**	399.5	1.8	0.766	0.004	**100.0**	570.4	2.0	0.708	0.003
**-2.0**	397.9	0.7	0.773	0.002	**110.0**	587.7	2.2	0.718	0.003
**0.0**	393.8	0.6	0.785	0.002	**120.0**	642.6	2.2	0.681	0.003
**0.5**	392.7	0.6	0.787	0.002	**130.0**	653.6	2.2	0.681	0.003
**1.0**	391.8	0.6	0.789	0.002	**140.0**	661.6	2.2	0.692	0.003
**1.5**	390.3	0.6	0.790	0.002	**150.0**	664.5	2.2	0.687	0.003
**2.0**	390.6	0.6	0.792	0.002	**160.0**	668.0	2.3	0.691	0.003
**2.5**	388.3	0.5	0.790	0.002	**180.0**	692.5	2.3	0.680	0.003
**3.0**	388.6	0.6	0.795	0.002	**200.0**	691.6	2.4	0.679	0.003
**3.5**	387.9	0.6	0.795	0.002	**220.0**	690.3	2.5	0.678	0.003
**4.0**	386.6	0.5	0.795	0.002	**240.0**	701.0	2.6	0.679	0.003
**4.5**	386.5	0.6	0.799	0.002	**260.0**	668.1	2.8	0.660	0.003
**5.0**	385.2	0.6	0.797	0.002	**280.0**	650.1	2.6	0.651	0.003
**5.5**	385.5	0.6	0.800	0.002	**300.0**	655.5	2.6	0.665	0.003
**6.0**	384.7	0.6	0.800	0.002	**320.0**	652.8	2.6	0.664	0.003
**6.5**	384.4	0.6	0.798	0.002	**340.0**	643.5	2.6	0.660	0.003
**7.0**	387.9	0.6	0.801	0.002	**350.0**	645.7	2.6	0.659	0.003
**7.5**	388.0	0.6	0.802	0.002	**400.0**	633.8	2.6	0.658	0.003
**8.0**	386.9	0.6	0.803	0.002	**450.0**	617.0	2.4	0.651	0.003
**8.5**	386.0	0.6	0.805	0.002	**500.0**	577.7	2.2	0.649	0.003
**9.0**	384.6	0.6	0.802	0.002	**550.0**	560.4	2.3	0.661	0.003
**9.5**	385.1	0.5	0.803	0.002	**600.0**	499.6	2.2	0.695	0.003
**10.0**	385.4	0.6	0.804	0.002	**650.0**	476.6	2.1	0.689	0.003
**12.0**	383.3	0.6	0.806	0.002	**700.0**	408.7	2.1	0.726	0.003
**14.0**	381.2	0.6	0.803	0.002	**750.0**	390.3	2.0	0.721	0.003
**16.0**	380.2	0.6	0.805	0.002	**800.0**	379.5	2.0	0.704	0.003
**18.0**	379.6	0.6	0.804	0.002	**850.0**	366.3	1.7	0.704	0.003
**20.0**	380.0	0.7	0.802	0.002	**900.0**	354.3	1.8	0.703	0.003
**22.0**	379.9	0.7	0.803	0.002	**950.0**	357.7	2.2	0.679	0.004
**24.0**	379.3	0.7	0.801	0.002	**1000.0**	313.0	2.3	0.708	0.004

Table S5. Magnetization relaxation times of $Dy_2@C_{80}(CF_3)$ diluted in polystyrene and measured at 6 K in different fields [a]

$\mu_0 H$, mT	τ, s	±	β	±	$\mu_0 H$, mT	τ, s	±	β	±
**-22.2**	1227.1	4.5	0.613	0.002	**3.3**	1183.6	4.1	0.624	0.003
**-22.2**	1230.9	4.5	0.604	0.003	**3.8**	1162.1	3.7	0.615	0.003
**-19.7**	1195.9	3.9	0.596	0.002	**4.3**	1128.6	3.5	0.605	0.002
**-19.7**	1206.3	4.3	0.606	0.002	**4.8**	1120.4	3.9	0.609	0.003
**-17.2**	1170.4	4.2	0.606	0.003	**5.3**	1103.1	3.7	0.606	0.003
**-17.2**	1226.8	4.5	0.624	0.003	**7.8**	1102.5	3.5	0.602	0.002
**-14.7**	1185.6	4.4	0.628	0.003	**8.8**	1159.4	4.1	0.632	0.003
**-14.7**	1139.4	3.8	0.593	0.003	**9.8**	1132.6	4.0	0.624	0.003
**-12.2**	1114.6	4.0	0.611	0.003	**10.8**	1092.1	3.8	0.610	0.002
**-12.2**	1125.9	4.0	0.610	0.002	**11.8**	1105.5	3.9	0.604	0.003
**-9.7**	1137.3	4.0	0.622	0.003	**12.8**	1119.6	3.9	0.603	0.003
**-9.2**	1103.6	3.7	0.604	0.003	**13.8**	1120.3	4.0	0.608	0.003
**-8.7**	1109.7	3.9	0.611	0.003	**14.8**	1139.9	4.1	0.612	0.002
**-8.2**	1096.4	3.7	0.613	0.002	**15.8**	1153.5	4.1	0.609	0.002
**-7.7**	1154.8	4.0	0.628	0.003	**16.8**	1165.7	4.2	0.610	0.002
**-7.2**	1126.4	3.7	0.603	0.003	**17.8**	1174.6	4.0	0.597	0.002
**-6.7**	1123.0	3.6	0.600	0.002	**22.8**	1227.1	4.5	0.614	0.002
**-6.2**	1109.1	3.8	0.611	0.003	**27.8**	1264.3	4.3	0.602	0.002
**-5.7**	1088.6	3.6	0.611	0.002	**32.8**	1284.5	5.0	0.618	0.002
**-5.2**	1149.0	3.9	0.624	0.003	**37.8**	1300.6	5.0	0.620	0.002
**-4.7**	1179.2	4.1	0.632	0.002	**42.8**	1304.2	5.2	0.623	0.002
**-4.2**	1132.8	3.8	0.616	0.003	**47.8**	1315.6	4.7	0.608	0.002
**-3.7**	1168.8	3.9	0.620	0.003	**52.8**	1349.1	5.5	0.637	0.002
**-3.2**	1226.2	4.4	0.642	0.003	**57.8**	1302.7	5.1	0.621	0.002
**-2.7**	1197.9	4.3	0.625	0.003	**62.8**	1298.0	4.9	0.608	0.002
**-2.2**	1219.0	4.4	0.630	0.002	**67.8**	1326.6	5.4	0.630	0.002
**-1.7**	1257.9	4.4	0.630	0.002	**72.8**	1289.4	4.7	0.605	0.002
**-1.2**	1290.7	4.5	0.622	0.002	**77.8**	1278.1	4.9	0.610	0.002
**-1.0**	1309.1	4.9	0.645	0.002	**82.8**	1271.4	4.7	0.602	0.002
**-0.8**	1202.0	4.3	0.624	0.003	**87.8**	1258.8	4.9	0.608	0.002
**-0.6**	1168.2	4.0	0.612	0.003	**92.8**	1250.4	4.9	0.607	0.002
**-0.4**	1114.8	3.7	0.607	0.002	**97.8**	1246.2	4.8	0.599	0.002
**-0.2**	826.5	2.9	0.572 [b]	0.002	**117.8**	1261.9	5.5	0.627	0.002
**0.0**	451.5	4.5	0.446 [b]	0.003	**137.8**	1174.0	4.8	0.605	0.002
**0.2**	727.1	2.3	0.544 [b]	0.002	**157.8**	1135.4	4.8	0.596	0.003
**0.4**	1117.3	3.5	0.603	0.002	**177.8**	1158.2	5.0	0.611	0.003
**0.6**	1168.4	3.8	0.611	0.002	**197.8**	1076.0	4.6	0.587	0.003
**0.8**	1179.2	4.2	0.618	0.003	**247.8**	1074.3	4.8	0.610	0.003
**1.3**	1333.3	4.9	0.649	0.002	**297.8**	970.8	4.3	0.581	0.003
**1.8**	1261.0	4.6	0.637	0.003	**347.8**	914.8	3.7	0.564	0.003
**2.3**	1214.5	4.4	0.628	0.003	**397.8**	847.9	3.6	0.571	0.003
**2.8**	1243.6	4.5	0.634	0.003					

[a] Magnetic field is corrected by 2.2 mT; some of the points were measured twice;

[b] Note the sharp decrease of β near QTM resonance in zero field; it can be caused by a large difference between QTM and thermal relaxation times, intrinsic QTM time is probably shorter.

AC measurements of $Dy_2@C_{80}(CF_3)$

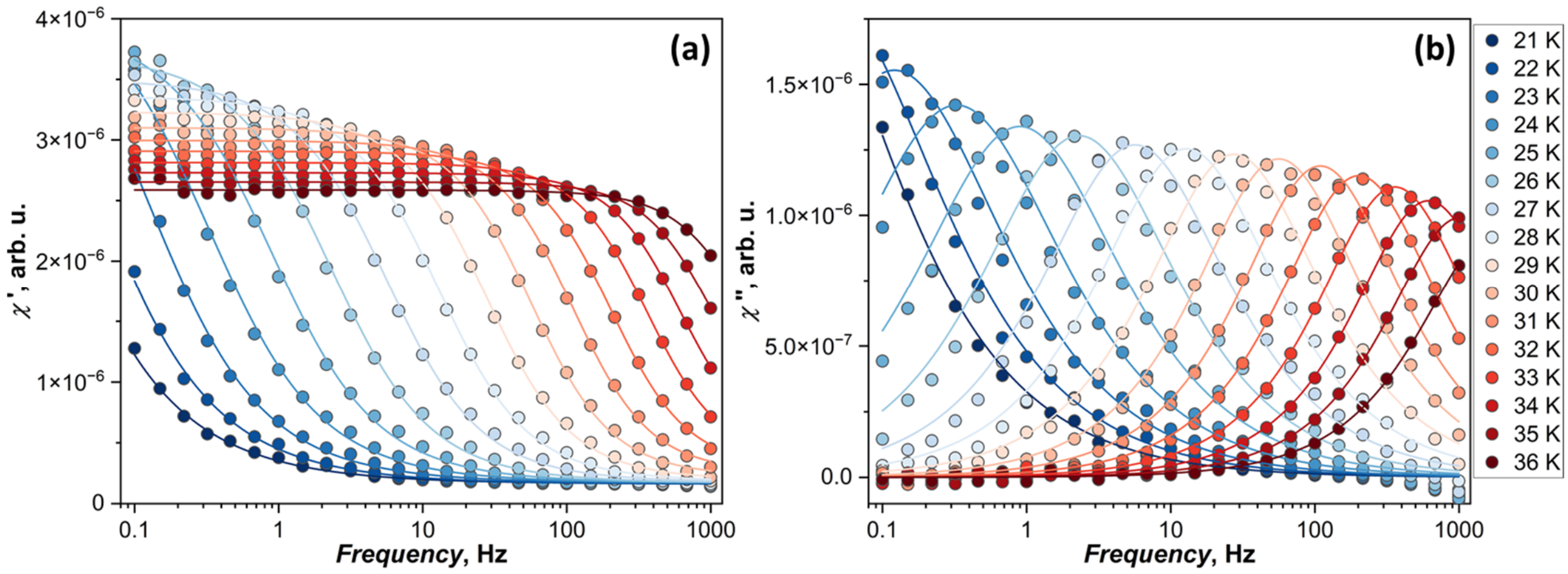

Figure S12. AC measurements of in-phase (χ', left) and out-of-phase (χ") magnetic susceptibility of $Dy_2@C_{80}(CF_3)$ at 0 T, dots are experimental data, lines are fitting with generalized Debye model.

Table S6. Magnetization relaxation times (τ) of $Dy_2@C_{80}(CF_3)$ from AC measurements

T, K	τ, s	±	α	±
23	1.32E+00	9.27E-02	0.257	0.014
24	4.87E-01	2.07E-02	0.243	0.013
25	1.78E-01	4.91E-03	0.218	0.011
26	6.88E-02	1.52E-03	0.192	0.010
27	2.82E-02	5.65E-04	0.170	0.010
28	1.24E-02	2.13E-04	0.150	0.009
29	5.79E-03	9.62E-05	0.132	0.008
30	2.83E-03	4.52E-05	0.108	0.008
31	1.46E-03	2.60E-05	0.091	0.010
32	7.87E-04	1.32E-05	0.081	0.009
33	4.43E-04	8.07E-06	0.066	0.009
34	2.60E-04	7.28E-06	0.052	0.012
35	1.56E-04	8.34E-06	0.045	0.017
36	8.59E-05	1.16E-05	0.064	0.026

Relaxation of magnetization in $[Dy_2@C_{80}]^-$

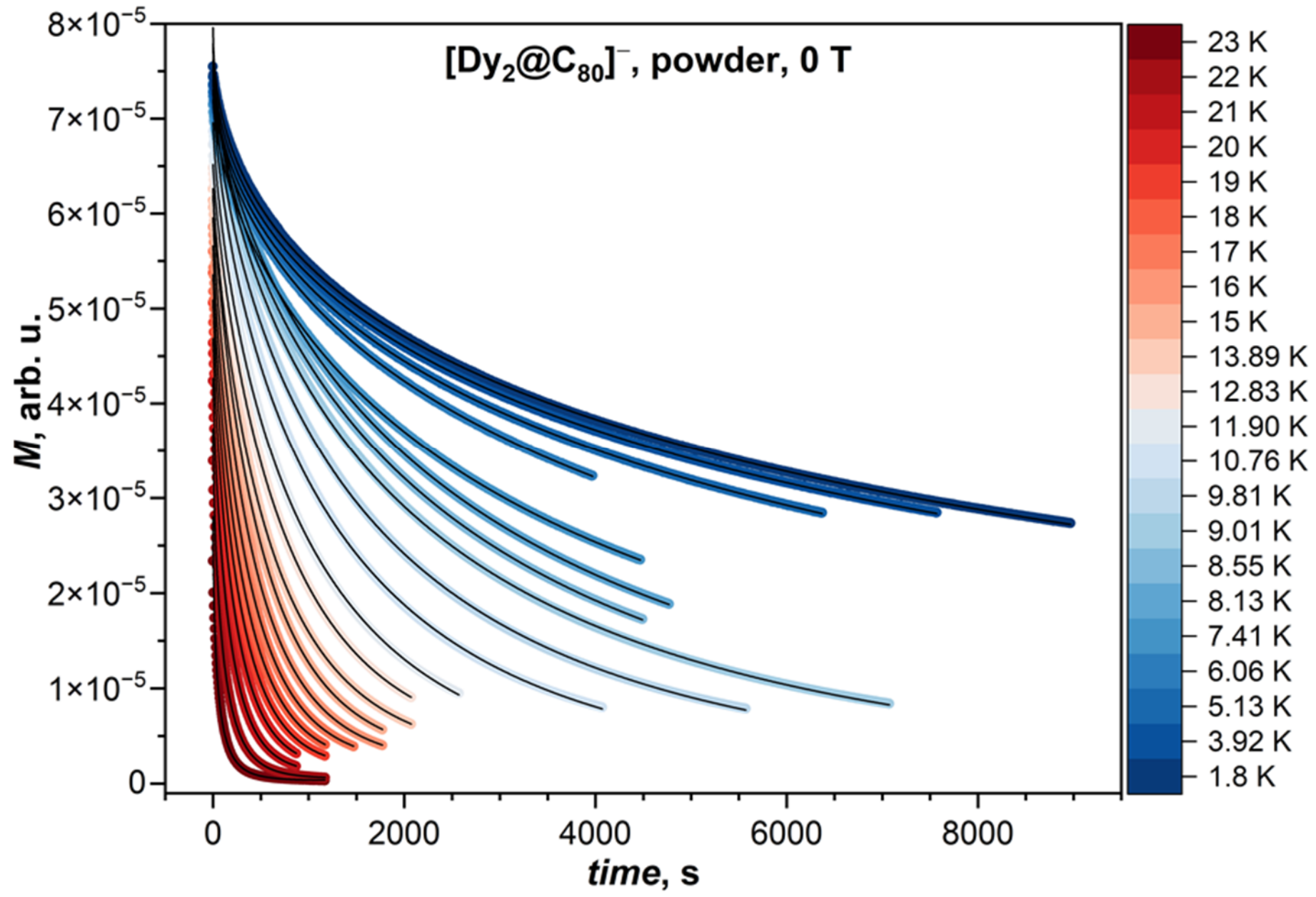

Figure S13. Selected magnetization decay (colored dots) and fitting with stretched exponential (black lines) of powder $[TBA]^+[Dy_2@C_{80}]^-$ measured at different temperatures in zero field.

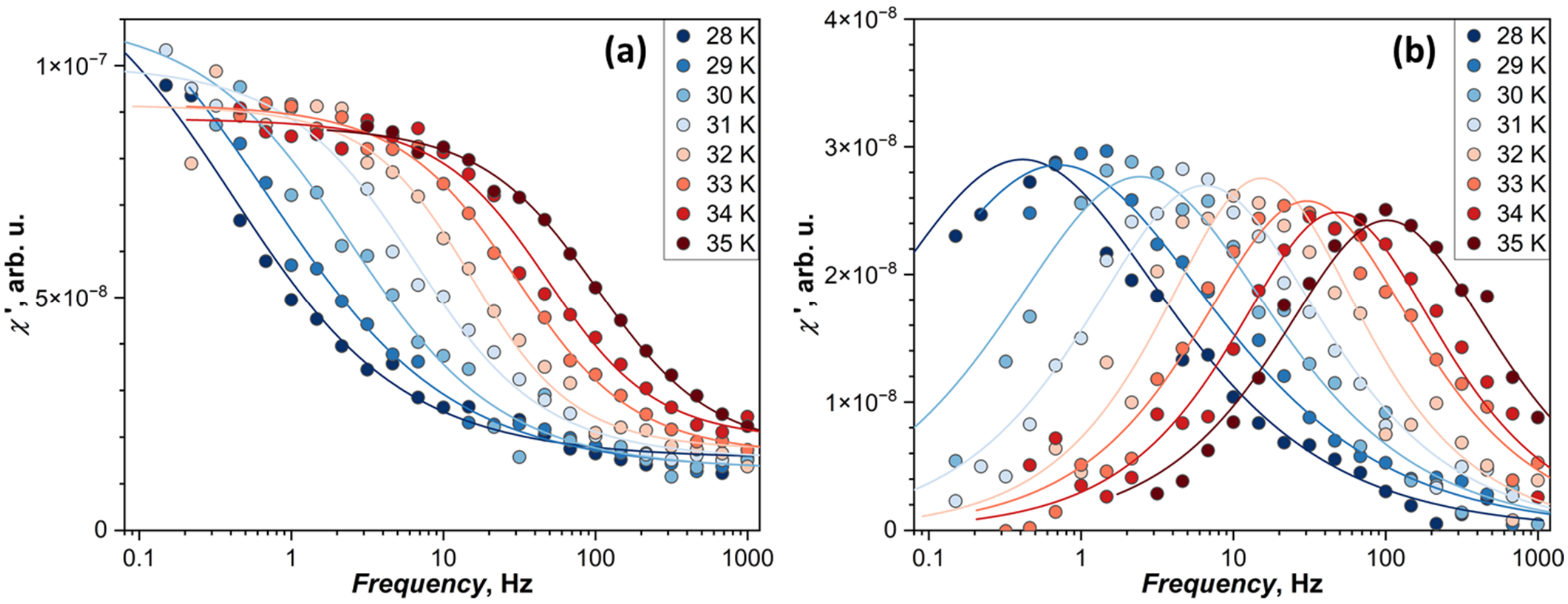

Figure S14. AC measurements of in-phase (χ′, left) and out-of-phase (χ″) magnetic susceptibility of $[Dy_2@C_{80}]^-$ at 0 T, dots are experimental data, lines are fitting with generalized Debye model.

Table S7. Magnetization relaxation times of $[Dy_2@C_{80}]^-$ from zero-field DC measurements at different temperatures

T, K	*τ*, s	±	β	±	*T*, K	*τ*, s	±	β	±
**1.8**	7652.0	10.9	0.477	0.001	**14.5**	575.5	0.4	0.862	0.001
**2.02**	7162.1	17.1	0.447	0.001	**15**	525.5	0.4	0.861	0.001
**2.3**	7380.9	13.0	0.467	0.001	**15.5**	478.7	0.5	0.855	0.001
**2.67**	7276.5	12.7	0.466	0.001	**16**	439.2	0.6	0.851	0.001
**3.17**	7163.7	12.2	0.468	0.001	**16.5**	401.8	0.8	0.843	0.002
**3.92**	7109.9	8.2	0.494	0.001	**17**	372.1	0.6	0.858	0.002
**4.45**	6838.8	6.4	0.509	0.001	**17.5**	343.5	0.7	0.852	0.002
**5.13**	6311.1	4.5	0.531	0.000	**18**	318.5	0.4	0.869	0.002
**6.06**	5249.6	2.5	0.582	0.000	**18.32**	292.9	0.3	0.872	0.001
**7.41**	3639.8	0.9	0.654	0.000	**18.65**	279.6	0.4	0.873	0.002
**8.13**	2871.1	1.0	0.672	0.000	**19**	270.9	0.6	0.858	0.002
**8.55**	2519.8	1.2	0.689	0.000	**19.32**	249.4	0.3	0.874	0.002
**8.77**	2258.1	3.7	0.664	0.001	**19.66**	235.1	0.4	0.873	0.002
**9.01**	2091.5	3.8	0.670	0.001	**20**	225.1	0.5	0.875	0.002
**9.26**	1935.9	3.8	0.677	0.001	**20.24**	206.4	0.4	0.870	0.002
**9.53**	1785.1	3.8	0.683	0.001	**20.49**	194.2	0.4	0.866	0.002
**9.81**	1645.2	3.8	0.690	0.001	**20.74**	180.5	0.5	0.855	0.002
**10.1**	1513.5	3.7	0.696	0.001	**21**	168.6	0.7	0.831	0.003
**10.42**	1399.5	3.6	0.704	0.001	**21.24**	152.7	0.4	0.870	0.004
**10.76**	1278.6	3.4	0.711	0.001	**21.49**	140.5	0.4	0.865	0.003
**11.11**	1174.2	3.1	0.722	0.001	**21.74**	126.5	0.5	0.842	0.004
**11.5**	1071.9	2.9	0.733	0.001	**22**	102.0	1.4	0.719	0.005
**11.9**	981.9	2.5	0.747	0.001	**22.24**	102.5	0.5	0.820	0.004
**12.35**	893.1	2.0	0.763	0.001	**22.49**	89.2	0.5	0.794	0.004
**12.83**	765.8	0.3	0.836	0.001	**22.74**	79.6	0.5	0.790	0.004
**13.34**	689.5	0.3	0.838	0.001	**23**	49.0	1.8	0.606	0.008
**13.89**	618.3	0.4	0.838	0.001					

In fitting decay curves with stretched exponential between 1.8 and 12.35 K, y_0 was measured separately and fixed during the fit. At higher temperatures, y_0 was fitted as free parameter.

Table S8. Magnetization relaxation times (τ) of $[Dy_2@C_{80}]^-$ from AC measurements

T, K	*τ*, s	±	α	±
28	3.87E-01	6.7E-02	0.396	0.031
29	2.24E-01	4.9E-02	0.425	0.032
30	6.53E-02	8.0E-03	0.346	0.038
31	2.46E-02	1.5E-03	0.281	0.024
32	1.04E-02	9.3E-04	0.189	0.044
33	5.24E-03	2.3E-04	0.240	0.020
34	3.31E-03	2.4E-04	0.209	0.033
35	1.56E-03	8.5E-05	0.235	0.024

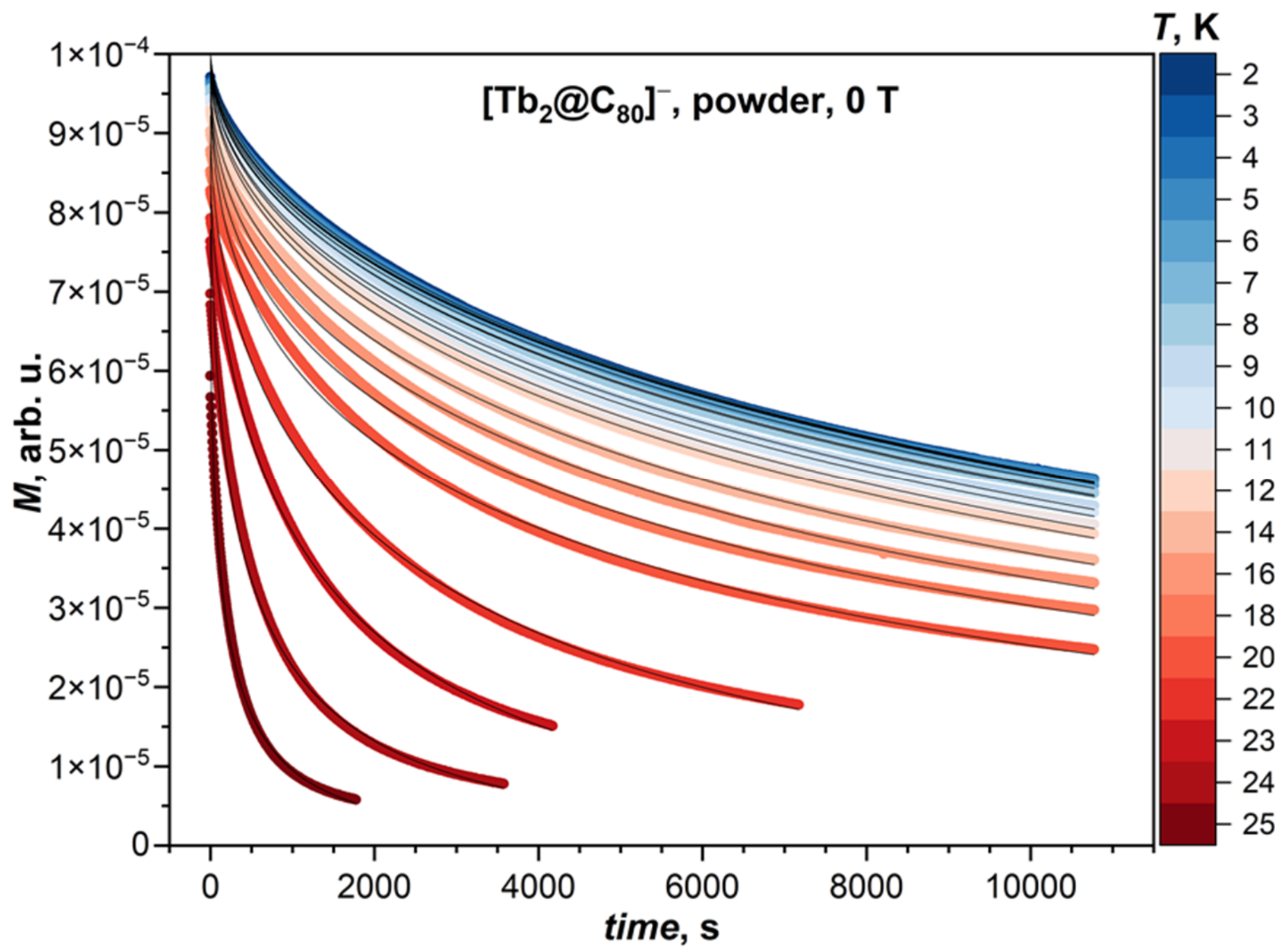

Figure S15. Selected magnetization decay (colored dots) and fitting with stretched exponential (black lines) of powder $[TBA]^+[Tb_2@C_{80}]^-$ measured at different temperatures in zero field.

Table S9. Magnetization relaxation times of $[Tb_2@C_{80}]^-$ from zero-field DC measurements at different temperatures

T, K	*τ*, s	±	β	±	*T*, K	*τ*, s	±	β	±
**2**	16518.6	16.1	0.558	0.001	**15**	9573.7	33.6	0.512	0.002
**2.25**	16000.1	16.0	0.559	0.001	**16**	8754.1	37.5	0.505	0.002
**2.5**	16371.8	16.3	0.555	0.001	**17**	7656.8	42.6	0.477	0.002
**3**	16665.3	16.8	0.556	0.001	**18**	7160.9	43.7	0.477	0.002
**3.5**	16432.0	16.9	0.553	0.001	**19**	6155.8	45.3	0.467	0.002
**4**	16670.7	16.7	0.556	0.001	**20**	5101.0	41.7	0.462	0.002
**5**	16589.4	16.7	0.556	0.001	**21**	4229.0	25.4	0.534	0.002
**6**	16277.0	16.8	0.556	0.001	**22**	2659.6	21.7	0.534	0.002
**7**	15932.9	17.1	0.557	0.001	**23**	1512.8	13.6	0.585	0.003
**8**	15294.1	18.3	0.553	0.001	**24**	740.9	7.0	0.629	0.004
**9**	14574.5	19.5	0.550	0.001	**24.5**	477.7	5.5	0.633	0.005
**10**	13582.2	20.1	0.547	0.001	**25**	287.5	5.2	0.604	0.006
**11**	12993.8	22.0	0.543	0.001	**25.5**	146.0	6.1	0.513	0.008
**12**	12136.5	24.2	0.539	0.001	**26**	67.1	5.5	0.423	0.010
**13**	11134.0	28.3	0.526	0.002	**26.5**	51.8	4.7	0.427	0.012
**14**	10531.1	28.9	0.525	0.002					

In fitting decay curves with stretched exponential, y_0 was measured separately and fixed during the fit.

Broken-symmetry DFT calculations

The strength of exchange coupling in Gd_2 dimetallofullerenes was calculated using broken-symmetry (BS) DFT approach at the PBE0-DKH level with SARC-DKH-TZVP basis set for Gd and SARC-adapted def2-TZVP basis for other elements. For each molecule, three BS states, in which spin was flipped for each of the Gd ions and for the unpaired electron residing on the Gd-Gd bonding orbital, were calculated. Examples of spin densities in the ground state and different BS states are shown in Fig. S16. To obtain coupling constants, the energies were mapped onto the spin Hamiltonian:

$$\hat{H}_{\mathrm{spin}} = -2j_{\mathrm{Gd-Gd}}\hat{S}_{\mathrm{Gd}'}\hat{S}_{\mathrm{Gd}''} - 2\hat{s}\left(K'_{\mathrm{Gd}-e}\hat{S}_{\mathrm{Gd}'} + K''_{\mathrm{Gd}-e}\hat{S}_{\mathrm{Gd}''}\right)$$

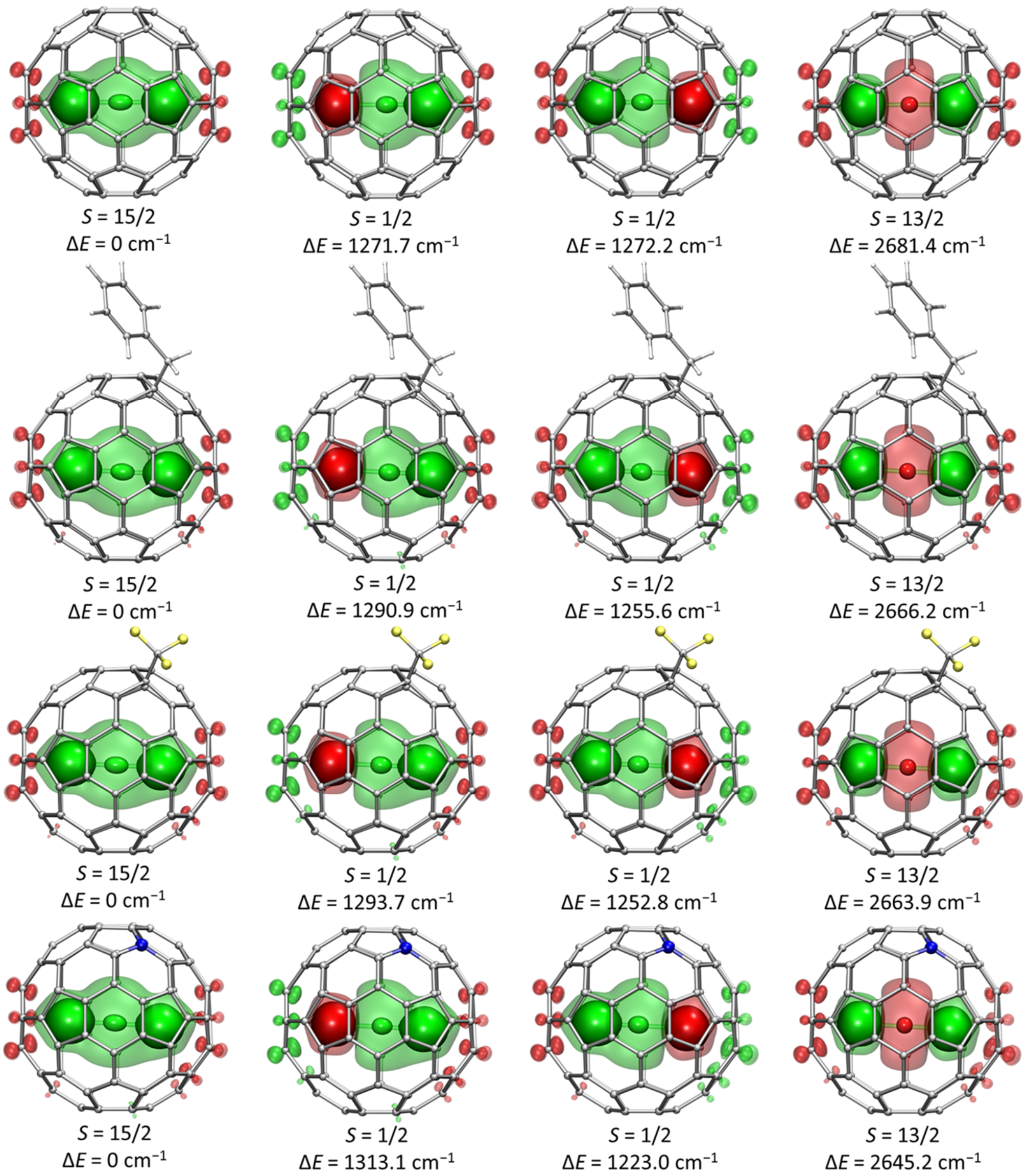

Figure S16. Spin densities and relative energies in the ground state and BS states with flipped spin of one of the Gd ions or unpaired electron. From top to bottom: $[Gd_2@C_{80}]^-$, $Gd_2@C_{80}(CH_2Ph)$, $Gd_2@C_{80}(CF_3)$, and $Gd_2@C_{79}N$. Transparent and solid spin-density isosurfaces are plotted at ±0.0012 a.u. and ±0.015 a.u., respectively; green – positive, red – negative spin density.

Table S10a. Single-ion LF splitting (cm^{-1}) computed for Dy di-EMFs at the CASSCF/RASSI-SO level

	$[Dy_2@C_{80}]^-$	$Dy_2@C_{80}(CH_2Ph)$	$Dy_2@C_{80}(CF_3)$	$Dy_2@C_{79}N$
KD1	0.0	0.0	0.0	0.0
KD2	286.6	280.6	213.0	181.4
KD3	419.0	408.4	382.1	371.0
KD4	538.7	522.6	539.1	541.1
KD5	627.5	622.0	640.6	653.7
KD6	747.0	742.5	731.0	734.5
KD7	864.3	856.1	831.4	820.1
KD8	934.5	923.3	896.9	887.8

Table S10b. Single-ion LF splitting (cm^{-1}) computed for Tb di-EMFs at the CASSCF/RASSI-SO level

	$[Tb_2@C_{80}]^-$	$Tb_2@C_{80}(CH_2Ph)$	$Tb_2@C_{80}(CF_3)$	$Tb_2@C_{79}N$
1	0.0	0.0	0.0	0.0
2	0.0	0.0	0.0	0.0
3	233.6	239.2	235.9	258.2
4	233.8	239.5	236.1	258.4
5	473.1	478.1	471.8	486.7
6	474.4	479.5	473.0	487.3
7	668.3	667.5	663.2	672.1
8	704.7	696.1	692.9	681.5
9	821.0	801.5	802.9	802.8
10	843.3	827.3	828.0	831.0
11	888.9	861.3	865.4	885.9
12	940.1	935.1	941.1	962.9
13	947.7	939.0	946.4	974.0

Table S11. Stevens factors θ_k of rank 2, 4, and 6 for Tb and Dy

	M = Tb	M = Dy
J, *M^{3+} multiplet*	6, $^7F_6$	15/2, $^6H_{15/2}$
g_J	1.5	4/3
θ_2×10^{-2}	−1.0101	−0.635
θ_4×10^{-4}; $\theta_4 J^4/\theta_2 J^2$	1.224; −0.44	−0.592; 0.52
θ_6×10^{-6}; $\theta_6 J^6/\theta_2 J^2$	−1.121; 0.14	1.035; −0.52

References

1. Mueller, U.; Förster, R.; Hellmig, M.; Huschmann, F. U.; Kastner, A.; Malecki, P.; Pühringer, S.; Röwer, M.; Sparta, K.; Steffien, M.; et al. The macromolecular crystallography beamlines at BESSY II of the Helmholtz-Zentrum Berlin: Current status and perspectives. *Eur. Phys. J. Plus* **2015**, *130* (7), 141.
2. Kabsch, W. XDS. *Acta Cryst. D* **2010**, *66* (2), 125-132.
3. Sparta, K. M.; Krug, M.; Heinemann, U.; Mueller, U.; Weiss, M. S. XDSAPP2.0. *J. Appl. Crystallogr.* **2016**, *49* (3), 1085-1092.
4. Sheldrick, G. Crystal structure refinement with SHELXL. *Acta Cryst. C* **2015**, *71* (1), 3-8.
5. Neese, F.; Wennmohs, F.; Becker, U.; Riplinger, C. The ORCA quantum chemistry program package. *J. Chem. Phys.* **2020**, *152* (22), 224108.
6. Neese, F. Software update: the ORCA program system, version 4.0. *WIREs Comput. Mol. Sci.* **2018**, *8* (1), e1327.
7. Dolg, M.; Stoll, H.; Savin, A.; Preuss, H. Energy-adjusted pseudopotentials for the rare earth elements. *Theor. Chim. Acta* **1989**, *75* (3), 173-194.
8. Yang, J.; Dolg, M. Valence basis sets for lanthanide 4f-in-core pseudopotentials adapted for crystal orbital ab initio calculations. *Theor. Chem. Acc.* **2005**, *113* (4), 212-224.
9. Weigend, F.; Ahlrichs, R. Balanced basis sets of split valence, triple zeta valence and quadruple zeta valence quality for H to Rn: Design and assessment of accuracy. *Phys. Chem. Chem. Phys.* **2005**, *7*, 3297-3305.
10. Pantazis, D. A.; Neese, F. All-Electron Scalar Relativistic Basis Sets for the Lanthanides. *J. Chem. Theory Comput.* **2009**, *5* (9), 2229-2238.
11. Aquilante, F.; Autschbach, J.; Baiardi, A.; Battaglia, S.; Borin, V. A.; Chibotaru, L. F.; Conti, I.; Vico, L. D.; Delcey, M.; Galván, I. F.; et al. Modern quantum chemistry with [Open]Molcas. *J. Chem. Phys.* **2020**, *152* (21), 214117.
12. Chibotaru, L. F.; Ungur, L. Ab initio calculation of anisotropic magnetic properties of complexes. I. Unique definition of pseudospin Hamiltonians and their derivation. *J. Chem. Phys.* **2012**, *137* (6), 064112.
13. Yang, W.; Velkos, G.; Sudarkova, S.; Büchner, B.; Avdoshenko, S. M.; Liu, F.; Popov, A. A.; Chen, N. Carbon cage isomers and magnetic Dy⋯Dy interactions in $Dy_2O@C_{88}$ and $Dy_2C_2@C_{88}$ metallofullerenes. *Inorg. Chem. Front.* **2022**, *9* (22), 5805-5819.
